# RAF kinases are stabilized and required for dendritic cell differentiation and function

**DOI:** 10.1038/s41418-019-0416-4

**Published:** 2019-09-20

**Authors:** Kristina Riegel, Janine Schlöder, Marco Sobczak, Helmut Jonuleit, Bernd Thiede, Hansjörg Schild, Krishnaraj Rajalingam

**Affiliations:** 1 Cell Biology Unit, University Medical Center Mainz, JGU-Mainz, Mainz, Germany; 2 Department of Dermatology, University Medical Center Mainz, JGU-Mainz, Mainz, Germany; 3 Department of Biosciences, University of Oslo, Oslo, Norway; 4 Institute of Immunology, University Medical Center Mainz, JGU-Mainz, Mainz, Germany

**Keywords:** Biochemistry, Cell biology, Immunology

## Abstract

RAF kinases (ARAF, BRAF, and CRAF) are highly conserved enzymes that trigger the RAF-MEK1/2-ERK1/2 (MAPK) pathway upon activation of RAS. Despite enormous clinical interest, relatively little is known on the role of RAFs in mediating immune responses. Here, we investigated the role of RAF kinases and MEK1/2 in dendritic cells (DCs), the central regulators of T cell-mediated antitumor immune responses and the adaptive immune system. We demonstrate that RAF kinases are active and stabilized at their protein levels during DC differentiation. Inhibition of RAF kinases but not MEK1/2 impaired the activation of DCs in both mice and human. As expected, DCs treated with RAF inhibitors show defects in activating T cells. Further, RAF and MEK1/2 kinases are directly required for the activation and proliferation of CD4^+^ T cells. Our observations suggest that RAF and MEK1/2 have independent roles in regulating DC function that has important implications for administering RAF–MAPK inhibitors in the clinics.

## Introduction

Kinases constitute the major component of the druggable genome and the development of novel inhibitors against kinases has been intensified because of their clinical success in treating a wide variety of malignancies [1]. The classical MAPK pathway encompassing RAF-MEK1/2-ERK1/2 has garnered enormous clinical interest as this pathway is triggered by oncogenic RAS and is required for tumorigenesis [2, 3]. RAF kinases are the first discovered RAS effectors and comprise three isoforms ARAF, BRAF, and CRAF of which BRAF is found often mutated in nearly 7% of human cancers [4]. This MAPK cascade is activated in response to growth factors and cytokines that control pivotal cellular processes like proliferation, migration, differentiation, and cell survival [5]. In 2011, the FDA approved the BRAF inhibitor vemurafenib for the treatment of unresectable or metastatic melanoma harboring a BRAFV600E mutation [6]. In the absence of BRAF-activating mutations, RAF inhibitors trigger paradoxical activation of MAPK cascade by promoting dimerization of RAF isoforms [7, 8]. To circumvent this issue, next generation RAF inhibitors which either block RAF dimerization or inhibit both the monomeric and dimeric RAF proteins were developed [9]. Further, several genomics-based studies revealed that patients acquire secondary mutations in their tumors leading to the reactivation of MAPK signaling and thus drug resistance [10, 11]. However, synergistic therapeutic regimes involving checkpoint inhibitors and vemurafenib have been shown to exhibit favorable clinical responses [12, 13].

While most of these studies focus on the role of RAF kinases in regulating tumorigenesis, relatively little is known about the role of RAFs in controlling innate and adaptive immune responses. Cancer cells evade immune surveillance through a variety of mechanisms and the current aim of immune therapeutics is to revoke the dampened immune system to effectively mount T cell-mediated killing of tumor cells [14, 15]. Dendritic cells (DCs) are professional antigen-presenting cells and their activation is required to initiate a profound T cell-mediated cancer immunity [16, 17]. Here, we investigated the role of RAF kinases in the regulation of DC differentiation and activation. We unveil that RAF kinases are stabilized and are required for DC activation and function. Intriguingly, we demonstrate that RAFs and MEK1/2 exhibit a cell type specific role in the regulation of DCs and CD4^+^ T cells.

## Results

**Fig. 1:**
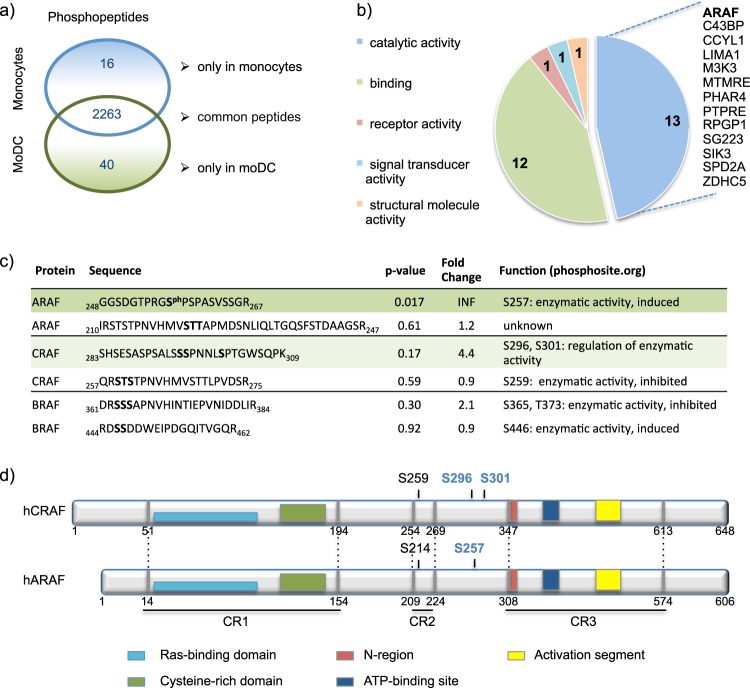
Phosphoproteome analysis of monocytes and moDCs. **a** Mass spectrometry-based proteome analysis was performed to compare the phosphoproteome of human monocytes with moDCs. Monocytes were isolated from buffy coats and cultured for 24 h in X-VIVO-15 medium supplemented with 1% heat-inactivated plasma. MoDCs were obtained through culturing monocytes with GM-CSF/IL-4 for 5 days. The Venn diagram shows phosphopeptides attributed to monocytes or moDCs detected in all three biological replicates of the respective samples. **b** The corresponding proteins of the phosphopeptides, detected exclusively in moDCs, were classified using Panther Classification System with the settings “GO:Slim Molecular Function” covering 28 proteins including ARAF. **c** Phosphopeptides of ARAF, BRAF, and CRAF are presented, which were identified in all three replicates of either monocytes or moDCs. Potential phosphorylation sites of the phosphorylated peptides are shown in bold. Among the identified phosphopeptides, the ARAF phosphopeptide covering amino acids 248–267 was the only one being significantly upregulated in moDCs. The CRAF phosphopeptide covering amino acids 283–309, which was found to be mono- and diphosphorylated, was higher in moDCs, but not significantly enriched. **d** The ARAF and CRAF phosphopeptides described in **c**, are located between the conserved regions CR2 and CR3

With the aim to identify differentially regulated factors during DC differentiation, we performed a hypothesis-free phosphoproteome analysis of human monocytes and monocyte-derived DCs (moDCs). From three biological replicates, we identified 2319 unique phosphopeptides of which 40 phosphopeptides representing 37 proteins were exclusively detected in moDCs (Fig. 1a). Analysis of these identified proteins with the Panther Classification System led to the identification of ARAF among other enzymes within the protein class “catalytic activity” (Fig. 1b). We then specifically evaluated the phosphopeptides from RAF isoforms (ARAF, BRAF, and CRAF) and detected a significant enrichment of phosphorylation of ARAF at S257 in moDCs (Fig. 1c). Further, a single CRAF mono- and diphosphorylated peptide with the potential phosphorylation sites S295, S296, and S301 was also slightly enhanced in moDCs (*p*-value = 0.17). Interestingly, the CRAF S296 phosphorylation site corresponds to ARAF S257 and they are located between the CR2 and CR3 regions [18] (Fig. 1d). According to previous studies, phosphorylation of ARAF at S257 correlates with an induced enzymatic activity [18]. However, phosphorylation at CRAF S296 and S301 was shown to have contradictory effects on the enzymatic activity depending on the mutants employed [19–21]. Further, S296 of CRAF is recognized as an ERK1/2 feedback phosphorylation site [21]. In contrast to the CRAF S296A/S301A mutant, overexpression of CRAF S296D/S301D mutant in HEK293T cells resulted in the activation of MEK1/2, suggesting that phosphorylation at these sites might be required for enzymatic activity (Supplementary Fig. 1a). A kinase assay with the ARAF mutants S257A and S257D revealed that both mutants are capable of phosphorylating the substrate MEK1, although the kinase activity of the ARAF S257A mutant is slightly lower (Supplementary Fig. 1c).

**Fig. 2:**
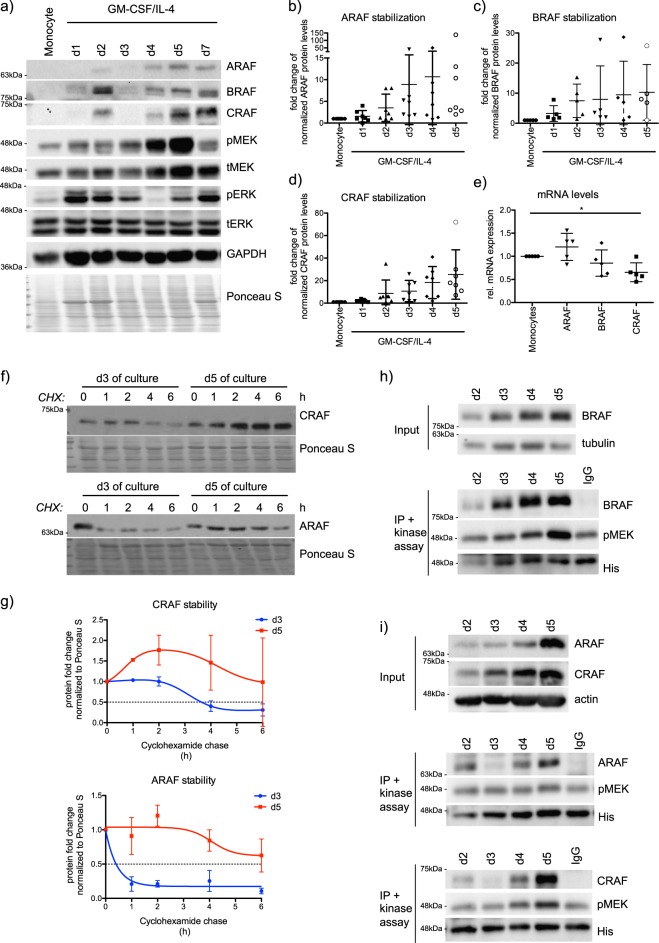
RAF kinases are stabilized during moDC differentiation. **a** Representative western blots monitoring the MAPK signaling during the differentiation of monocytes to moDCs induced by GM-CSF/IL-4. **b** The change in total ARAF (*n* = 7), **c** BRAF (*n* = 5) and **d** CRAF levels (*n* = 7) was quantified during the progressive differentiation to moDCs. **e** Real-time PCR analysis was performed to compare mRNA levels of A−/B− and CRAF from monocytes with immature moDCs (*n* = 5). **f** Protein stability of ARAF and CRAF in cells on day 3 and day 5 of differentiation was investigated by cycloheximide (CHX) chase experiments and subsequent western blot analysis. **g** The half-life of CRAF and ARAF was determined by quantifying the decrease in total protein levels observed in the CHX experiments relative to Ponceau S staining (*n* = 3). **h** Kinase activity of BRAF immunoprecipitated from cells differentiated for 2–5 days with GM-CSF/IL-4 was studied as mentioned in the methods. Phosphorylation of the kinase dead MEK K97A substrate at S217/S221 was investigated by western blot. Shown is a representative experiment. **i** Same as in **h**, but with precipitated ARAF and CRAF

Since these data suggest that ARAF and CRAF kinases are probably active in moDCs, we then checked for the activation dynamics of the MAPK signaling components during moDC differentiation by immunoblot analysis. Interestingly, we detected a steady and striking increase in the protein levels of ARAF, BRAF, and CRAF during the course of moDC differentiation (Fig. 2a–d). The upregulation of RAF protein levels was not due to transcriptional regulation, as their mRNA levels remained largely unchanged between human monocytes and moDCs (Fig. 2e). Cycloheximide chase experiments were performed with cells on day 3 and day 5 of differentiation in order to evaluate the half-life of ARAF and CRAF (Fig. 2f). Quantification of three independent experiments revealed an enhanced half-life of ARAF and CRAF in immature moDCs on day 5 (Fig. 2g). Treatment with MG132, a proteasomal inhibitor, caused especially for ARAF a significant, but small increase in the protein levels in cells differentiated to moDCs for 3 days, but not in cells differentiated to moDCs for 5 days (Supplementary Fig. 2a, b). While a similar trend was observed with CRAF (Supplementary Fig. 2a, c), MG132 treatment did not cause significant changes in BRAF protein levels (Supplementary Fig. 2a, d). Further, we have not detected any significant changes in the polyubiquitination of endogenous ARAF and CRAF (Supplementary Fig. 2e). These data suggest that especially in moDCs (obtained after 5 days of differentiation) other protein quality control machineries apart from the proteasome may possibly play a role in the regulation of RAF proteostasis.

In contrast to the stabilized RAF proteins, we failed to detect any consistent changes in MEK1/2 protein levels (Supplementary Fig. 3a) as well as in the activating phosphorylation status of MEK1/2 (Supplementary Fig. 3b). We detected alteration in the activation dynamics of ERK1/2 during the differentiation process, but failed to detect any major changes in the ERK1/2 phosphorylation in moDCs when compared with the monocytes (Supplementary Fig. 3c). These data indicate that the strong increase in the protein levels of RAF proteins is probably not translated into the activation of MEK1/2 and ERK1/2.

To directly test whether RAF kinases are activated during differentiation of DCs, we performed a kinase assay with endogenous RAF proteins immunoprecipitated during moDC differentiation (from day 2 to day 5). In case of BRAF, the amount of precipitated protein increased comparable with protein levels seen in the input (Fig. 2h). As expected, the increasing BRAF levels translated to a higher phosphorylation of the inactive MEK substrate. CRAF-induced MEK phosphorylation was especially detected in precipitates from day 5 of culture, while ARAF-induced MEK phosphorylation was only slightly higher compared with the background phosphorylation observed in the IgG control sample (Fig. 2i). Consequently, RAF kinases are stabilized and activated in moDCs and the higher activity corroborated with enhanced protein amounts. It is worth mentioning, that the increase in protein levels of RAF proteins was also seen during the differentiation of murine bone marrow (BM) cells to bone marrow-derived dendritic cells (BMDC) (Supplementary Fig. 3d).

**Fig. 3:**
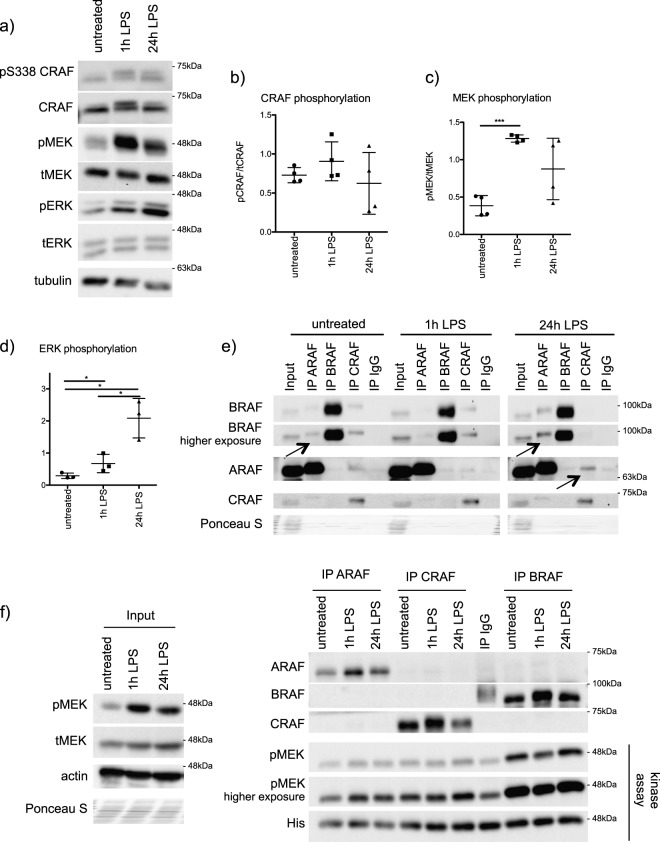
RAF kinases are dimerized in moDCs in response to LPS. **a** Immature moDCs were stimulated for 1 and 24 h with LPS (100 ng/ml) and phosphorylation of CRAF (S338), MEK (S217/S221), and ERK1/2 (Y202/Y204) was determined by western blot analysis. **b** The relative level of CRAF (*n* = 3), **c** MEK1/2 (*n* = 4), and **d** ERK1/2 phosphorylation (*n* = 3) was determined by the ratio of phosphorylated protein to total protein levels. **e** Endogenous RAF proteins were immunoprecipitated from immature moDCs and from LPS-stimulated moDCs (100 ng/ml, 1 h and 24 h). Immunoprecipitates were analyzed by western blot. The data are representative for four independent experiments. Arrows are highlighting the observed RAF-interactions. **f** Kinase activity of RAF proteins immunoprecipitated from immature moDCs and LPS-stimulated moDCs (100 ng/ml, 1 h and 24 h) was studied as mentioned in the “methods” section. Phosphorylation of the kinase dead MEK K97A substrate at S217/S221 was investigated by western blot and a representative experiment is shown

As RAF kinases are stabilized during moDC differentiation, we tested if they are further activated in response to extracellular signals like lipopolysaccharide (LPS). For the following experiments, cells obtained after 5 days of differentiation are referred to as immature moDCs. Stimulation of immature moDCs with LPS led to a strong increase in the activating phosphorylation of MEK1/2 (S217/S221) and ERK1/2 (Y202/Y204) and to a minor increase in CRAF (S338) phosphorylation (Fig. 3a–d). As RAF kinases dimerize for their activation [22], we performed interaction studies at endogenous levels. In immature moDCs, BRAF was already detected in ARAF immunoprecipitates suggesting the presence of RAF heterodimers (Fig. 3e). Interestingly, 24 h of LPS stimulation induced a stronger interaction between BRAF and ARAF (Fig. 3e). Further, ARAF was also detected in CRAF precipitates from moDCs stimulated for 24 h with LPS (Fig. 3e). We then performed a kinase assay with endogenous RAF proteins immunoprecipitated from immature moDCs and LPS-stimulated moDCs (1 h and 24 h) (Fig. 3f). While all three RAF kinases were constitutively active in immature moDCs, the kinetics of RAF activation upon LPS stimulation followed different kinetics than the observed LPS-induced MEK1/2 phosphorylation. For instance, a strong increase in MEK1/2 phosphorylation was detected after 1 h of LPS stimulation, while CRAF and BRAF activities decreased after 1 h of LPS stimulation and then recovered again after 24 h of LPS stimulation. These data suggest that LPS-induced MEK phosphorylation is possibly RAF-independent in moDCs.

We then set out to investigate the role of RAF kinases in moDCs by employing loss of function studies with siRNAs. As RAF kinases are stabilized and form complexes with each other, we performed various combinations of double knockdowns (ARAF + BRAF, ARAF + CRAF, and BRAF + CRAF) in moDCs. We achieved a modest but reproducible knockdown of ~30% of both RAF isoforms (Supplementary Fig. 4a, b) and as expected, we could not detect any substantial effects on the activation of moDCs as measured by the expression of surface markers (Supplementary Fig. 4c). However, a double knockdown of ARAF and CRAF as well as BRAF and CRAF led to a small but significant reduction in the secretion of the pro-inflammatory cytokine IL-12p70 upon LPS stimulation (Supplementary Fig. 4d). Our attempts to establish CRISPR-based approaches to establish reproducible knockouts in human moDCs failed. We then resorted to a variety of RAF inhibitors to further delineate the role of RAF kinases in regulating moDC function. In particular, we employed the pan-RAF inhibitor LY3009120 (Supplementary Fig. 5a) as its antitumor activity has been successfully shown in in vivo BRAF^mut^ and KRAS^mut^ colorectal cancer xenograft models [23]. LY3009120 blocks monomeric and dimeric RAF proteins with equal efficiency and has been developed to circumvent the issue of RAF inhibitor mediated paradoxical MAPK activation [24].

We confirmed that LY3009120 inhibited the MAPK pathway in at least three cancer cell lines (HeLa, Calu1, and MDA-MB231) at a concentration of 1 μM (Supplementary Fig. 5b, c). Further, LY3009120 significantly reduced the viability of several tumor cell lines at 1 μM concentration (Supplementary Fig. 5d). We then confirmed the efficiency of the inhibitor by performing in vitro kinase assays with CRAF and BRAF immunoprecipitated from moDCs treated with LY3009120 (Supplementary Fig. 5e).

**Fig. 4:**
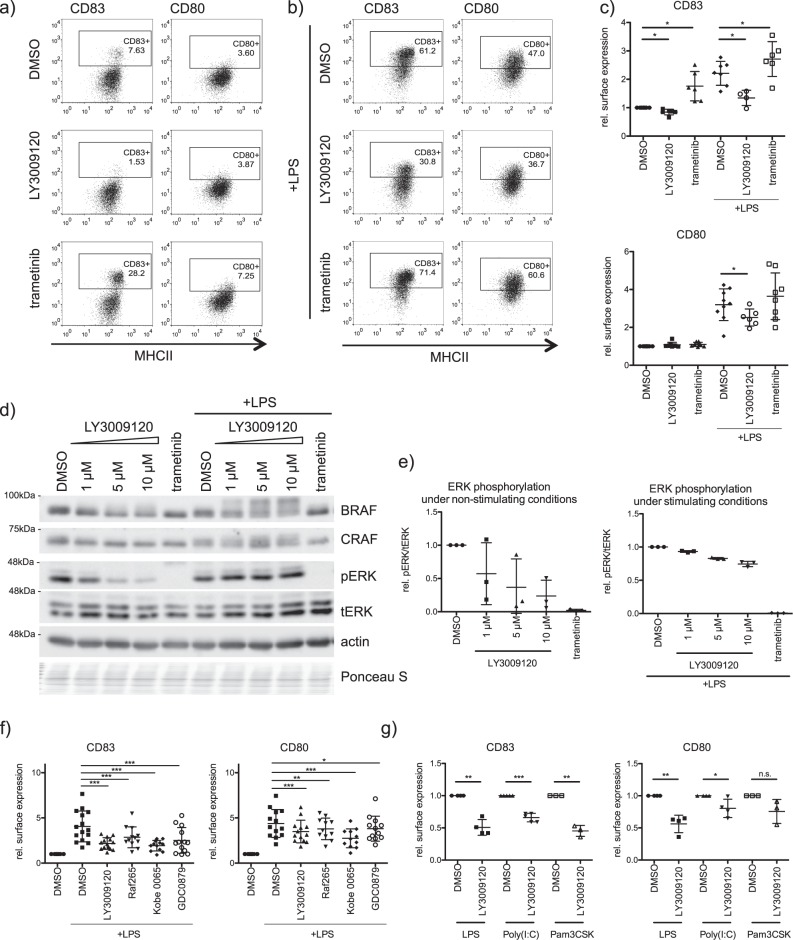
Role of RAF kinases in the regulation of moDC activation. **a** Surface markers of moDCs (CD83 and CD80) were analyzed by flow cytometry after 48 h treatment with trametinib (1 μM) or with LY3009120 (1 μM) in absence or **b** presence of LPS (100 ng/ml). **c** The relative mean fluorescence of the surface markers of multiple independent experiments was quantified. **d** Immature moDCs were treated for 6 h with LY3009120 (1, 5, and 10 µM) or trametinib (1 µM) in presence or absence of LPS (100 ng/ml). ERK1/2 (Y202/Y204) phosphorylation was investigated by western blot and **e** the relative phosphorylation levels were quantified by the ratio of phosphorylated protein to total protein (*n* = 3). **f** CD80 and CD83 surface expression was determined by flow cytometry after treating moDCs for 48 h with other RAF Inhibitors (LY3009120: 1 µM, *n* = 14; Raf265: 0.5 μM, *n* = 12, Kobe: 10 μM, *n* = 12; GDC-0879: 1 μM, *n* = 13) in presence of LPS and **g** after the treatment of moDCs with Poly(I:C) (binds to TLR3, 50 µg/ml, *n* = 4) or Pam3Cys (binds to TLR1/2, 1 µg/ml, *n* = 3) in presence of LY3009120. Shown are relative mean fluorescence intensities. (*n.s.* not significant)

**Fig. 5:**
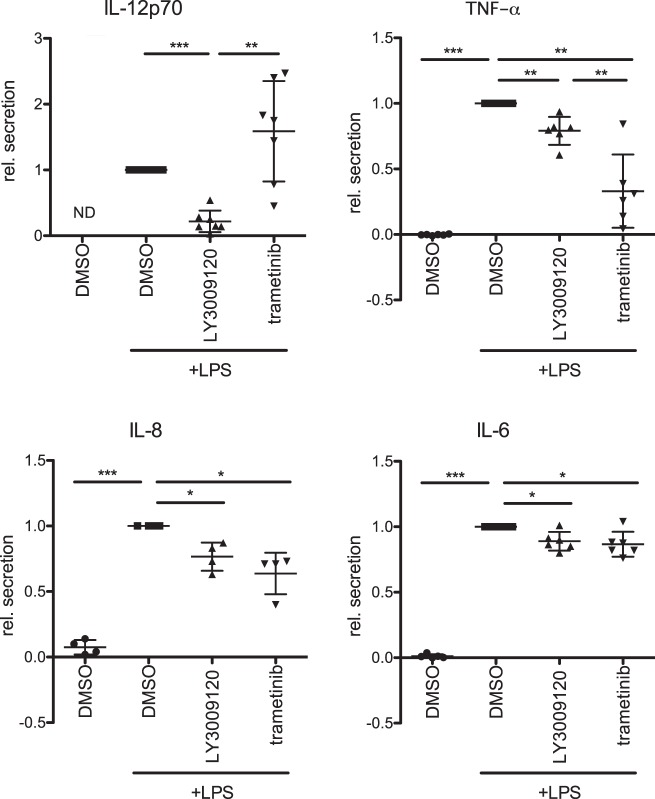
Role of RAF kinases in the regulation of moDC cytokine secretion. The secretion of the inflammatory cytokines IL-12p70 (*n* = 7), IL-6 (*n* = 6), TNF-α (*n* = 6), and IL-8 (*n* = 4) was studied by ELISA after 48 h treatment with trametinib (1 μM) or with LY3009120 (1 μM) in presence of LPS (100 ng/ml). MoDCs treated only with DMSO served as control. (*ND not detected*)

We then challenged moDCs with RAF and MEK1/2 inhibitors. Surprisingly, MEK1/2 inhibition by trametinib did not compromise LPS-induced upregulation of the activation markers CD80 and CD83, while RAF inhibition significantly reduced their surface expression (Fig. 4a–c). Intriguingly, the effects on MHCII and CD86 surface expression levels were less strong (Supplementary Fig. 6a, b). Dose response studies suggested that 1 μM concentration of LY3009120 already lead to a saturation in the reduction of the activation markers in moDCs (Supplementary Fig. 6c). Interestingly, while increasing LY3009120 concentrations inhibited the MAPK pathway under steady state conditions, LPS stimulation induced ERK1/2 phosphorylation despite the block of RAF kinases in moDCs (Fig. 4d, e). In contrast, trametinib, a well-studied and clinically approved MEK1/2 inhibitor, inhibited phosphorylation of ERK1/2 under both stimulating and nonstimulating conditions (Fig. 4d, e). The inhibitory effect of RAF inhibition on surface expression of moDCs was further confirmed with multiple RAF inhibitors (LY3009120, GDC-0879, RAF265, and Kobe 0065) (Fig. 4f and Supplementary Fig. 6d). We also tested if CD80 and CD83 surface expression is affected in Poly(I:C)- or Pam3CSK4- stimulated moDCs (Supplementary Fig. 6e). As shown in Fig. 4g, inhibition of RAF kinases led to similar effects when the cells were activated with these TLR3 and TLR1/TLR2 agonists, respectively. In addition, we further checked the cytokine profile of moDCs treated with RAF inhibitors. LPS-induced secretion of IL-6, IL-8, and TNF-α was significantly reduced after RAF or MEK1/2 inhibition (Fig. 5). LY3009120 and trametinib showed contrary effects on IL-12p70 secretion as RAF inhibition reduced and MEK1/2 inhibition rather promoted its secretion. Taken together, these data suggested that RAF and MEK1/2 might have independent roles in regulating moDC activation.

**Fig. 6:**
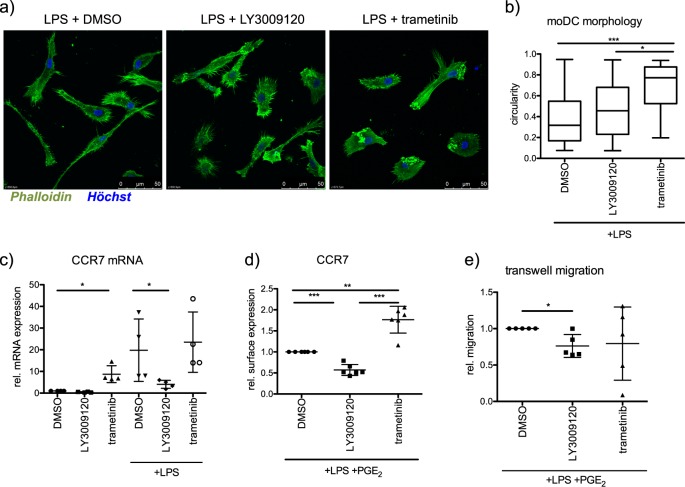
Role of RAF kinases in moDC migration. **a** F-actin of LPS-stimulated moDCs, which were treated with LY3009120 (1 µM) or trametinib (1 µM), were stained with phalloidin and visualized by a Leica SP8 confocal microscope. **b** Morphology was analyzed by determining the circularity index of 26–60 cells per condition. **c** CCR7 mRNA levels were determined by real-time PCR after treating moDCs for 48 h with LY3009120 (1 µM) or trametinib (1 µM) in the presence of LPS (*n* = 4). **d** The surface marker expression of CCR7 was analyzed by flow cytometry after treating moDCs with LY3009120 (1 µM) or trametinib (1 µM) while stimulating with LPS (100 ng/ml) and PGE_2_ (1 µg/ml) (*n* = 6). **e** Directed migration of moDCs towards the cytokine CCL21 (200 ng/ml) was determined in transwell migration experiments by counting successfully migrated cells after 3 h (*n* = 5)

Upon activation, DCs migrate to T cell rich zones in the draining lymph nodes to finally induce the activation and clonal expansion of antigen-specific naive T cells [25]. As RAF-MAPK signaling contributes to cell polarity, cell shape, and migration [26–28], we initially tested whether RAF or MEK1/2 inhibition elicited morphological changes on the moDC phenotype. Indeed, we detected that moDCs exhibited a more rounded morphology after inhibition of MEK1/2 or RAFs by trametinib or LY3009120, respectively (Fig. 6a, b). Furthermore, RAF inhibition had a negative effect on the mRNA expression of the chemokine receptor CCR7, which directs mature DCs to the draining lymph nodes [29] (Fig. 6c). While LPS alone induced a modest surface expression of CCR7, stimulation of moDCs by a combination of LPS and PGE_2_ enhanced the same (Supplementary Fig. 7). However, inhibition of RAF prevents the surface expression of CCR7 despite stimulation with LPS and PGE_2_. Interestingly, under the same settings, inhibition of MEK1/2 enhanced CCR7 expression (Fig. 6d). The reduced CCR7 surface expression is probably contributed by the reduction in the mRNA levels of CCR7 after LY3009120 treatment (Fig. 6c). As expected, migration of moDCs towards CCL21 was inhibited by RAF inhibitor treatment, while trametinib treatment did not show any significant effects (Fig. 6e).

**Fig. 7:**
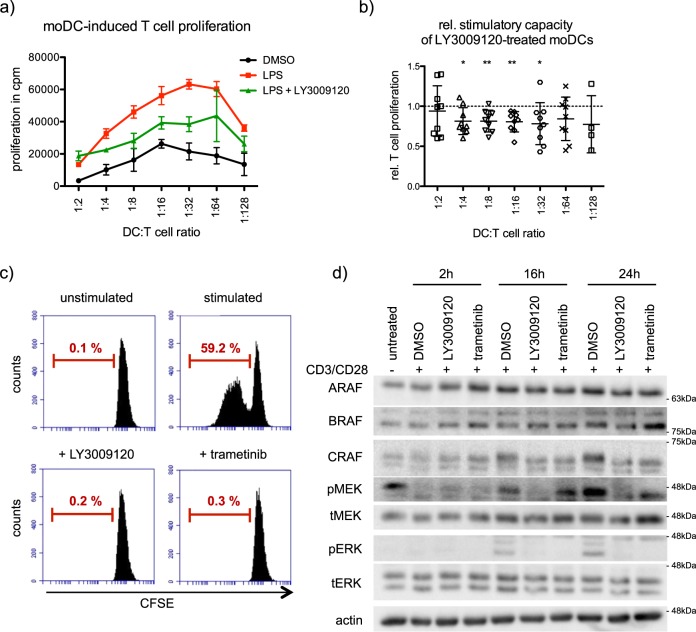
RAFs are required for activation of moDCs and T cells. **a** To evaluate moDC induced T cell proliferation, an MLR with moDCs stimulated with LPS in presence of LY3009120 (1 µM) and allogeneic CD4^+^ cells was performed. T cell proliferation was determined via ^3^H-thymidine incorporation. Shown is a representative experiment (*n* = 9). Error bars show standard deviation of three technical replicates. **b** The induced T cell proliferation of moDCs treated with LY3009120 (1 µM) was determined relatively to the corresponding stimulated control (*n* = 9). **c** CFSE-labeled CD4^+^ T cells stimulated with 0.5 µg/ml anti-CD3 mAb and 1 µg/ml anti-CD28 mAb were simultaneously treated with LY3009120 (1 μM) or trametinib (1 μM). After 4 days, dividing cells were identified by flow cytometry. The data are representative of three independent experiments. **d** MAPK signaling of stimulated CD4^+^ T cells treated for 0, 2, 16, and 24 h with LY3009120 (1 μM) or trametinib (1 μM) was analyzed by western blot

As RAF kinases are required for the activation and migration of moDCs, we performed functional assays by employing co-culture experiments including allogeneic CD4^+^ T cells. RAF inhibitor treated moDCs induced a significantly lower CD4^+^ T cell proliferation compared with control moDCs (Fig. 7a, *n* = 9). The negative effect of RAF inhibitors on CD4^+^ T cell priming was evident when DCs and T cells were co-cultured in ratios between 1:4 and 1:32 (Fig. 7a, b). Interestingly, RAF and MEK1/2 inhibition directly blocked CD4^+^ T cell proliferation when stimulated by CD3/CD28 monoclonal antibodies (Fig. 7c). Consistent with the published observations, these data suggest that MEK1/2 and RAFs are functioning through the MAPK cascade in controlling T cell proliferation [30, 31]. Furthermore, treatment with both RAF and MEK1/2 inhibitors prevented the activation of ERK1/2 kinases (Fig. 7d).

**Fig. 8:**
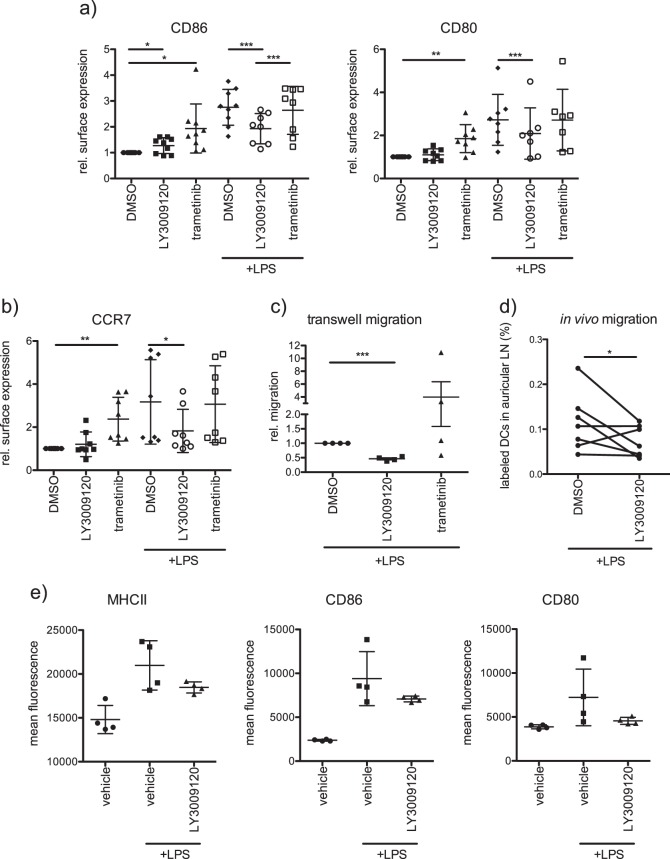
Role of RAFs on murine DC activation and migration. **a** BMDCs from C57BL/6 mice were treated with trametinib (1 μM) or with LY3009120 (1 μM) in presence or absence of LPS (100 ng/ml) for 48 h and surface marker expression (CD86, and CD80) was analyzed by flow cytometry. Shown are relative mean fluorescence intensities of multiple independent experiments. **b** Expression of the chemokine receptor CCR7 was investigated as described in **a**. **c** In vitro migration towards the cytokine CCL21 (200 ng/ml) was investigated with a transwell migration chamber. Successfully migrated BMDCs were determined after 3 h by MTT assay (*n* = 4). **d** In vivo migration of labeled BMDCs towards auricular lymph nodes was monitored and quantified in mice as mentioned in the methods part (*n* = 7). **e** The in vivo effect of LY3009120 was investigated on splenic DCs from C57BL/6J mice. The inhibitor (15 mg/kg; solved 0.5% CMC) was administered i.p. on day 0 and day 1. LPS (10 µg/mouse) was injected i.p. on day 2. Control mice received the corresponding vehicle. Six hours after LPS administration, spleens were isolated and surface expression of MHCII, CD86, and CD80 on DCs was analyzed by flow cytometry (*n* = 4)

As most of these observations were performed with human moDCs, we tested the effects of RAF inhibitors with murine BMDCs with an aim to extrapolate these observations to in vivo mouse models. Consistent with moDCs, LY3009120 treatment significantly reduced the surface expression of CD86 and CD80 in response to LPS (Fig. 8a and Supplementary Fig. 8a, b). Further, surface expression of CCR7 was reduced which in turn led to a decreased migration of murine BMDCs towards CCL21 (Fig. 8b, c). Consistent with these observations, the migration of labeled BMDCs towards auricular lymph nodes from the ear lobe of mice was significantly reduced upon RAF inhibitor treatment (Fig. 8d). Taken together, these data suggest that RAF kinases are required for the activation of both moDCs as well as murine BMDCs. Finally, we then evaluated the effects of LY3009120 on the activation of DCs in vivo by administering the drug intraperitoneally for 2 consecutive days (15 mg/kg), before LPS injection. RAF inhibitor treatment led to a reduction in the surface expression of MHCII, CD80, and CD86 from DCs isolated from spleen after 6 h of LPS stimulation (Fig. 8e and Supplementary Fig. 8c). We employed an RAF inhibitor concentration that has been shown to inhibit tumor growth in NU/NU mice [32]. These data indicate that pan-RAF inhibitors might inhibit the activation and function of DCs in vivo.

## Discussion

Despite the fact that RAF kinases were in the limelight owing to the clinical success of RAF inhibitors in treating a subset of human cancers, their role in regulation of the immune system is not well understood. Previous studies have shown that BRAF inhibition in melanoma cell lines resulted in a decreased secretion of immunosuppressive factors like IL-10, VEGF, and IL-6 and enhanced expression of melanocyte differentiation antigens (MDA) to improve recognition by MDA-specific T cells [33]. Further, an increased infiltration of CD8^+^ T cells into the tumor was observed after BRAF inhibition which is accompanied by an increased PD-L1 expression [12]. The study by Ott et al. demonstrated that the BRAF inhibitor PLX4032 did not have detrimental effects on human moDC function [34]. Owing to dimerization-induced paradoxical MAPK activation, the next generation of pan-RAF inhibitors were developed. Synergistic therapeutic regimes combining targeted and immune therapeutics are believed to be beneficial for the patients [35]. Thus, characterizing the role of RAF isoforms in the regulation of immune cells is clearly warranted for better clinical administration of pan-RAF inhibitors.

We performed an unbiased phosphoproteome analysis of monocytes vs. moDCs and identified several factors that are differentially regulated (Fig. 1). Here, we focused on elucidating the role of RAF kinases in the regulation of DCs, the sentinels of the innate immune system. We found that RAF kinases are stabilized during moDC differentiation and that they are active in moDCs (Figs. 1 and 2). The exact molecular machinery driving the proteostasis of RAF kinases in moDCs deserves further studies. Interestingly, we could not detect any significant alterations in the polyubiquitination of CRAF and ARAF (Supplementary Fig. 2) and our attempts to enrich ubiquitinated species of endogenous proteins from moDCs with UBAN domains showed no significant effects.

We uncover that apart from stabilization, especially ARAF and BRAF form heterodimers in moDCs and that stimulation induced interaction between ARAF and CRAF (Fig. 3). Nevertheless, only minor effects on CRAF phosphorylation can be observed upon LPS stimulation, while there is a strong increase in  MEK1/2 activation (Fig. 3). Further, kinase assays with endogenous RAF kinases revealed, that despite the constitutive activation of RAF kinases in immature moDCs, LPS stimulation results in different activation kinetics of RAF kinases compared with MEK activation (Fig. 3). Interestingly, while RAF inhibition reduced ERK1/2 activation under nonstimulating conditions, LPS stimulation was still sufficient to induce ERK1/2 phosphorylation despite the block of RAF kinases (Fig. 4). Consistent with the questioning if MEK1/2 is a direct substrate of RAF kinases in stimulated moDCs, inhibition of RAF and MEK1/2 kinases exhibit opposing effects on DC activation (Fig. 4). Unlike MEK1/2 inhibition, treatment with multiple RAF inhibitors reduced the activation of human and murine DCs in vitro (Figs. 4 and 8). Further, we also detected opposing effects with respect to the expression of CCR7 and migration of DCs towards CCL21 (Figs. 6 and 8). In addition, we detected that secretion of IL-12p70, a key cytokine responsible for the activation of CD4^+^ T cells and priming towards Th1 responses [36], is reduced in moDCs upon RAF inhibitor treatment (Fig. 5). Previous studies have shown that in immune cells MEK1/2 kinases can be activated in a RAF-independent but Tpl2-dependent manner [37–39]. Whether Tpl2 activation is required in moDCs under these settings needs further studies. Nevertheless, secretion of IL-6, TNF-α, and IL-8 were reduced by the treatment with RAF as well as MEK1/2 inhibitor in moDCs indicating that RAF proteins may not function exclusively in a MEK1/2-independent manner (Fig. 5). It has been already reported that MEK1/2 inhibition attenuates the secretion of these cytokines [39, 40]. In all these experiments, we employed concentrations that inhibited the growth of tumor cells in vitro (Supplementary Fig. 5) and in vivo [32]. When administered in vivo, RAF inhibitor reduced LPS-mediated activation of DCs in the spleen (Fig. 8). Whether the inhibitor treatment exhibits consistent effects between resident and migratory DCs in vivo is currently unclear. As expected, co-culture experiments with allogeneic T cells unveiled that the stimulatory capacity of RAF-inhibited moDCs was significantly reduced compared with the control group (Fig. 7). Interestingly, unlike in DCs, both RAF and MEK1/2 kinases are required for the proliferation of CD4^+^ T cells which suggests that the vectorial MAPK signaling is possibly intact in these cells.

Taken together, our study provides further insights into the role of RAF kinases in the activation and function of DCs and has clinical implications as pan-RAF inhibitors may have adverse effects on DC function. Further studies are required to explore how RAF activation is regulated in moDCs. Although it is well known, that GM-CSF-induced signaling leads to RAS activation among others [41], RAS activation upon LPS stimulation is not precisely understood [42]. Whether RAF kinases are regulated in an RAS-dependent manner and whether activation of RAF contributes to the enhanced protein stability of ARAF and CRAF deserves further studies.

## Methods

### Generation of human monocyte-derived dendritic cells (moDCs)

This study was conducted in accordance with the Declaration of Helsinki. Buffy coats were obtained from healthy volunteers at the University Medical Center Mainz with approval by the local ethical committee (Landesaerztekammer Rheinland-Pfalz). Peripheral blood mononuclear cells (PBMC) were isolated from buffy coats following standard procedures [43] and 1.5 × 10^7^ PBMCs were seeded in pre-warmed RPMI medium containing 1% autologous plasma per well of a six-well plate. The cells were allowed to adhere onto the plastic surface for 20 min at 37 °C. Nonadherent cells were removed by washing and the remaining adherent cells were differentiated into DCs in X-VIVO-15 medium (Cat. No. BE04-418Q, Lonza, Verviers, Belgium) supplemented with 1% autologous plasma, 400 IU/ml human GM-CSF (Leukine, Sanofi) and 200 IU/ml recombinant human IL-4 (Cat. No. 11340045, Immunotools) as described before [44]. After 2 days, 1 ml medium was replaced by 1 ml fresh X-VIVO-15 medium supplemented with 1% autologous plasma, 800 IU/ml GM-CSF and 200 IU/ml IL-4. Immature DCs were harvested on day 5 of culture and 1–2 × 10^6^ cells were seeded per well of a six-well plate in medium supplemented with 1% plasma, 400 IU/ml GM-CSF and 200 IU/ml IL-4 for further treatment.

### Generation of dendritic cells derived from murine bone marrow (BMDCs)

Bone Marrow (BM) of C57BL/6J mice was isolated by cutting the ends of femur and tibia and flushing the bone cavities with Phosphate Buffer Saline (PBS, pH 7.4), supplemented with 1% FCS using a 23G cannula. The BM was collected in a 50 mL tube and centrifuged at 1300 rpm for 10 min, followed by lysis of erythrocytes by a 2 min incubation in Greys lysis buffer. The reaction was stopped by adding PBS with 1% FCS. The cell suspension was filtered through a 40 µm cell strainer (Greiner Bio-one) and the obtained cells were resuspended in IMDM culture medium (Iscove’s Modified Dulbecco's Medium, 5% FCS, 2 mM l-glutamine, 1% sodium pyruvate). Cells were seeded in six-well nonadherent plates (3–6 × 10^6^/well) and culture medium was supplemented with 1% murine GM-CSF and 2.5 ng/ml recombinant murine IL-4 (Cat. No. 12340043, Immunotools). On day 3 and day 5, medium was exchanged to remove nonadherent cells and to supplement cultures with fresh medium containing GM-CSF and mIL-4. Immature DCs were harvested on day 7 of culture. For further treatment 2 × 10^6^ cells/well were seeded into nonadherent six-well plates and cultured in medium containing GM-CSF (1%) and IL-4 (2.5 ng/ml).

### Stimulation and treatment of DCs

To investigate the role of MAPK signaling in DCs of human and murine origin, inhibitors blocking components of the MAPK signaling were added to the cultures. LY3009120 (1 µM, Cat. No. S7842, Selleckchem), GDC-0879 (1 µM, Cat. No. S1104 Selleckchem), Raf265 (0.5 µM, Cat. No. S2161 Selleckchem), and Kobe005 (10 µM, Cat. No. S8303 Selleckchem) were employed to block RAF Kinases. Trametinib (1 µM, Cat. No. S2673 Selleckchem) was employed to block MEK1/2. In order to block proteasomal degradation, MG132 (10 µM, Merck Millipore) was added to the cultures for 6 h. All inhibitors were dissolved in DMSO unless otherwise stated, DCs were treated with the inhibitors in the presence or absence of LPS stimulation (100 ng/ml, Cat. No. L6143, Sigma) for 48 h and used for further analyses. Alternatively, DCs were stimulated with Pam3CSK4 (1 µg/ml, Cat. No. tlrl-pms, InvivoGen), Poly(I:C) (50 µg/ml, Cat. No. 27–4732–01, GE Healthcare) or with a combination of LPS and PGE_2_ (1 µg/ml, Cat. No. 14010, Cayman, solved in X-VIVO-15/10% ethanol).

### Immunoprecipitation

To immunoprecipitate endogenous proteins, 4–8 × 10^6^ DCs were lysed in 500–800 µl IP buffer [100 mM NaCl, 50 mM TrisCl, 1% NP40, 1 mM NaVO_3_, 1 mM NaF, and 1× protease inhibitor] for 30 min on ice. After clearing the lysates by centrifugation for 15 min at 14,000 rpm (at 4 °C), the protein concentration was determined using Pierce 660 nm Protein Assay Reagent (Cat. No. 22660, ThermoFisher Scientific). Endogenous CRAF, ARAF, or BRAF was immunoprecipitated from 200 to 300 µg total protein by overnight incubation (4 °C) with the target antibody and the subsequent precipitation of the antigen–antibody complexes by agarose-coupled protein A/G beads (Cat. No. 11–134–515–001 and 11–243–233–001, Roche). Beads were washed with IP buffer and bound proteins were analyzed by SDS-PAGE and immunoblotting.

### RAF kinase assay

Endogenous RAF proteins were immunoprecipitated from human immature moDCs, from moDCs stimulated with LPS (1 and 24 h) and from cells differentiated to moDCs for 2–5 days. Alternatively, RAF proteins were immunoprecipitated from moDCs treated for 6 h with DMSO or LY3009120. In both cases, IgG control was included. Further, kinase activity of V5-tagged ARAF WT and ARAF mutants (ARAF Y301D/Y302D, ARAF R362H, ARAF S257A, and ARAF S257D) was investigated. Constructs were overexpressed in HEK293T cells and immunoprecipitated via the V5 tag. The empty vector served as control. Agarose-coupled protein A/G beads were employed to precipitate the antibody/antigen complexes to which a reaction mix of 1× kinase buffer [10× buffer: 100 mM MgCl_2_, 250 mM β-glycerolphosphate, 250 mM HEPES pH 7.5, 50 mM benzamidine, 5 mM DTT, 10 mM NaVO_3_; diluted to 1× with H_2_O] and 1 µg of kinase dead His-MEK1 K97A (Cat. No. M02-16H, SignalChem) was added in a total volume of 38 µl. After adding MgATP (Enzo Life Sciences; stock: 20×; diluted to 1×), the reaction was incubated at 30 °C for 30 min. Kinase assays were stopped by adding 10 µl of Laemmli buffer and loaded onto an SDS-PAGE gel for immunoblot analysis.

### mRNA Isolation, cDNA synthesis and qPCR

RNA from 1–2 × 10^6^ cells was isolated by employing either RNA isolation kits (ThermoFisher and Roche) following the manufacturer’s instructions or by TRIzol RNA extraction. Therefore, cells were washed with PBS and lysed in 1 ml TRIzol (Ambion/ThermoFisher). Two hundred microliters of chloroform was added to these samples and vortexed for 15 s followed by 2 min incubation at room temperature. Further, the samples were centrifuged at 14,000 rpm for 15 min at 4 °C. The upper aqueous phase was transferred to a tube containing 0.5 ml isopropanol and incubated for 10 min at room temperature followed by another centrifugation step of 15 min at 14,000 rpm at 4 °C. The pellet formed by the isolated RNA was washed once with 75% ethanol, air-dried and resuspended in appropriate amount of ultrapure water. cDNA was synthesized from 1000 ng of the isolated RNA using the RevertAid First Strand cDNA Synthesis Kit (Cat. No. K1622, Thermo Scientific) and the supplied random hexamer primers.

All real-time PCR reactions were performed in triplicates on an iCycler (BioRad cxn96 or connect/Applied Biosystems Step One Plus) using EvaGreen (Cat. No. 27490, Axon). The mRNA levels of the housekeeping gene 18S were used for normalization and relative expression levels were calculated as ∆∆Ct.

Real-time PCR primers:

18S: 5′-agaaacggctaccacatcca-3′/3′-caccagacttgccctcca-5′

CRAF: 5′-acagatattctacacctcacg-3′/3′-aattgcatcctcaatcatcc-5′

ARAF: 5′-cccatcttgacaaaatctaagg-3′/3′-ccttgtctagagagtcgtag-5′

BRAF: 5′-atatctggaggcctatgaag-3′/3′-ctgaaagagatgaaggtagc-5′

CCR7: 5′-ttgtcattttccaggtatgc-3′/3′-aatgatggagtacatgataggg-5′

### Flow cytometry

In vitro generated and treated DCs were washed with PBS and stained for 30 min at 4 °C with the fluorescent-labeled antibodies listed in section “Antibodies”. To discriminate between live and dead cells, the cells were simultaneously treated with the Fixable Viability Dye 780 (Cat. No. 65–0865–14, eBioscience). If cells were stained for CCR7 expression the incubation with the staining solution was performed for 20 min at room temperature and 10 min at 4 °C. After two washing steps with PBS, samples were acquired on a BD FACSCanto II and data were analyzed with BD FACSDiva software (version 6.0) and FlowJo software. The mean fluorescence intensities (MFI) of independent experiments were quantified relatively to the corresponding controls (DCs treated with DMSO).

### Transwell migration assay

For transwell migration experiments, in vitro generated murine BM-derived DCs were treated for 16 h with LPS alone or with LPS in the presence of either the RAF inhibitor LY3009120 or the MEK1/2 inhibitor trametinib along with unstimulated control. In case of the human moDCs the stimulation was done with a combination of LPS and PGE_2_ as the addition of PGE_2_ led to an increased CCR7 expression.

After two washing steps with PBS, 4 × 10^5^ cells were resuspended in serum-free medium into a 5 µm transwell migration chamber (Cat. No. 3421, Corning). The lower chamber was filled with serum-free media supplemented with 200 ng/ml recombinant CCL21 (human: Cat. No. 11343180, Immunotools; murine: Cat. No. 250–13, PeproTech) as a chemoattractant. The cells were left to migrate for 3 h and the number of successfully migrated BMDCs were counted using TC 20TM automated cell counter (BioRad #145-0101), while the amount of migrated human DCs was determined with an MTT assay.

### Morphological analysis using phalloidin

A total of 1 × 10^6^ human moDCs were seeded into 12-well plates and were treated for 24 h with either DMSO, LY3009120 or trametinib in the presence or absence of LPS. Treated cells were resuspended and 200 µl of the cell suspension were transferred into a 12-well plate assembled with poly-L-lysine (Cat. No. P6282, Sigma) coated cover slips. The cells were allowed to adhere for 6 h. Subsequently, cells were washed twice with PBS, fixed with 4% PFA (Roti histofix) for 10 min and permeabilized with 0.1% Triton X-100 in PBS for 5 min at room temperature. After two additional washing steps, cells were blocked with 1% BSA in PBS for 1 h at room temperature. F-actin was stained using the high affinity probe phalloidin conjugated to green fluorescent Oregon Green 488 dye (Cat. No. 07466, ThermoFisher). Staining was performed with 4 U/ml phalloidin in blocking buffer for 20 min in the dark. After staining the nuclei with 2 µg/ml Hoechst for 5 min, cells where washed and mounted with Mowiol on cover slides. Cells were imaged using a Leica SP8 confocal microscope (63×, oil immersion objective, Oregon Green, excitation at 488 nm). The circularity of the cells was determined with ImageJ software.

### Enzyme-linked immunosorbent assay (ELISA)

Cytokine secretion of human moDCs was measured by ELISA according to the manufacturer’s instructions (BD Bioscience). After treating the DCs for 48 h with either DMSO, LY3009120 or trametinib in presence of LPS, the supernatant was collected and centrifuged for 10 min at 15,000 rpm and 4 °C. The supernatant of DCs treated with DMSO alone served as a negative control. The secretion of the pro-inflammatory cytokines IL-12p70, IL-6, IL-8, and TNF-α were determined.

### Mixed lymphocyte reaction

The ability of DCs to induce T cell proliferation was investigated with a mixed lymphocyte reaction. Human moDCs on day 5 were treated with DMSO or LY3009120 in the presence or absence of LPS for additional 48 h. Mature DCs were harvested, washed with PBS and resuspended in X-VIVO-15 medium at a cell density of 0.5 × 10^6^ cells/ml.

Simultaneously, CD4^+^ T cells were isolated from PBMCs of purified buffy coats from a different donor. The isolation of CD4^+^ T cells was accomplished by employing human CD4 microbeads (Cat. No. 130–045–101, Miltenyi Biotec) following the manufacturer’s instructions. CD4 microbeads were added in a ratio of 1 µl per 10^7^ cells. The isolation was performed by using MACS separation columns 25 LS and a Miltenyi magnet according to the manufacturer’s instructions. The enriched CD4^+^ T cells were washed with PBS and resuspended in X-VIVO-15 medium at a cell density of 1 × 10^6^ cells/ml.

The mixed lymphocyte reactions were done in triplicates in a 96-well plate. The highest DC:T cell ratio was 1:2 (5 × 10^4^ DCs: 1 × 10^5^ CD4^+^ cells). A twofold serial dilution was performed with DCs, which resulted in DC:T cell ratios ranging from 1:2 to 1:256. Controls containing DCs or CD4^+^ T cells alone were included. On day 4, ^3^H-thymidine was added to each well (37 kBq/well) and cells were cultured for an additional 16 h. DC-induced T cell proliferation was measured by ^3^H-thymidine incorporation using a liquid β-scintillation counter.

### T cell proliferation assay

The direct effect of LY3009120 and trametinib was investigated on CFSE-labeled CD4^+^ T cells, which were stimulated with 0.5 µg/ml anti-human CD3 mAb (clone OKT3, Bio X Cell) and 1 µg/ml anti-human CD28 mAb (clone: CD28.2, BD Pharmingen™). The dilution of CFSE fluorescence was recorded in the FITC channel on day 4.

### In vivo migration of BMDCs

Immature BMDCs were treated for 16 h with LY3009120 in presence of LPS. Following the manufacturer’s instructions, LY3009120-treated BMDCs were stained with eBioscience™ Cell Proliferation Dye eFluor™ 450 (ThermoFisher) and the corresponding control BMDCs were stained with eBioscience™ Cell Proliferation Dye eFluor™ 670 (ThermoFisher). A total of 0.5 × 10^6^ BMDCs were subcutaneously injected into the ear fold of 6 weeks old, male C57Bl/6J mice (volume: 20 µl). Each mouse received LY3009120-treated BMDCs in one ear, while the corresponding control BMDCs were injected into the other ear. After 48 h mice were sacrificed and the auricular lymph nodes were isolated. Lymph nodes were digested for 1 h at 37 °C in 1 mg/ml collagenase D (Cat. No. 11088866001, Sigma). After stopping the reaction with 0.5 mM EDTA, cells were passed through a cell strainer (40 µm) and analyzed by flow cytometer. The percentage of migrated per LN was determined.

### In vivo effect of LY3009120 on DCs

LY3009120 (15 mg/kg, dissolved in 0.5% carboxymethylcellulose) was i.p. injected into 6 weeks old, female C57Bl/6J on 2 consecutive days. LPS injection (10 µg/mouse) was done on day 2. One control group received exclusively the vehicle. The other control group received no inhibitor, but was treated with LPS. Four mice per condition were used. After 6 h LPS injection mice were sacrificed and the spleen was isolated. Organs were digested for 1 h at 37 °C in 1 mg/ml collagenase D (Cat. No. 11088866001, Sigma). After stopping the reaction with 0.5 mM EDTA, cells were passed through a cell strainer (40 µm) and stained for flow cytometric analysis.

### Statistical analysis

*P* values were obtained by *t*-test in GraphPad Prism and if not stated otherwise *p* < 0.05 was considered as a significant difference. Statistical significance levels are annotated as *P < 0.05, **P < 0.01, ***P < 0.001.

## Supplementary information

Supplementary Information

Supplementary Figures
