# Supplementary material for: RAF kinases are stabilized and required for dendritic cell differentiation and function
Source: Cell Death Differ. 2019 Sep 20;27(4):1300–15. doi: 10.1038/s41418-019-0416-4 (PMC7206131; doi:10.1038/s41418-019-0416-4)
Supplement: Supplementary file 1 — Supplementary Information [file 41418_2019_416_MOESM1_ESM.docx]

**Supplementary Information:**

**Supplementary methods**

## Phosphoproteome analysis

Monocytes were isolated from buffy coats and cultured for 24 h in X-VIVO-15 medium supplemented with 1% plasma. The moDCs were harvested on day 5 as described in the methods. Cells were washed with PBS, lysed in RIPA buffer [50 mM Tris-HCl (pH 7.5), 250 mM NaCl, 10% glycerol, 1% Triton X-100, 1 mM NaVO_3_, 1 mM NaF, protease inhibitor mixture (1:100 dilution)] for 30 min on ice, frozen at -80°C until further use.

The cell lysates were precipitated with six volumes of acetone overnight, centrifuged at 13000 rpm for 10 min and air dried. The protein pellet was dissolved in 200 µl of 6 M urea in 100 mM ammonium bicarbonate. The dissolved protein was mixed with 10 µl of 200 mM DTT in 0.1 M Tris-HCl, pH 8 and incubated at 30^o^C for 30 min. 30 µl of freshly prepared 200 mM iodoacetamide was added and the sample was incubated at room temperature in the dark for 1 h. Subsequently, 40 µl of 200 mM DTT was added and incubated at 30^o^C for 30 min. The sample was then diluted with 960 µl of 50 mM ammonium bicarbonate before 10 µg of trypsin GOLD (Promega, Madison, WI, USA) was added and incubated at 37^o^C for 16 h. The digestion was finally quenched by adding 20 µl formic acid (50%) and peptides were cleaned by SPE using a Strata C18-E cartridge (55 µm, 70 Å, Phenomenex, Værlose, Denmark). Phosphopeptides were enriched using TiO_2_ beads (Titansphere, TiO_2,_ GL Sciences Inc, Japan) as previously described^1^. The tryptic phosphopeptides were dissolved in 10 µl 0.1% formic acid/2% acetonitrile and 5 µl were analyzed using an Ultimate 3000 RSLCnano-UHPLC system connected to a Q Exactive mass spectrometer (Thermo Fisher Scientific, Bremen, Germany) equipped with a nano-electrospray ion source. For liquid chromatography separation, an Acclaim PepMap 100 column (C18, 2 µm beads, 100 Å, 75 μm inner diameter, 50 cm length) (Dionex, Sunnyvale CA, USA) was used. A flow rate of 300 nL/min was employed with a solvent gradient of 4-35% B in 180 min. Solvent A was 0.1% formic acid and solvent B was 0.1% formic acid/90% acetonitrile. The mass spectrometer was operated in the data-dependent mode to automatically switch between MS and MS/MS acquisition. Survey full scan MS spectra (from m/z 400 to 2,000) were acquired with the resolution R = 70,000 at m/z 200, after accumulation to a target of 1e6. The maximum allowed ion accumulation times were 60 ms. The method used allowed sequential isolation of up to the ten most intense ions, depending on signal intensity (intensity threshold 1.7e4), for fragmentation using higher-energy collisional induced dissociation (HCD) at a target value of 1e5 charges, NCE 28, and a resolution R = 17,500. Target ions already selected for MS/MS were dynamically excluded for 30 sec. The isolation window was m/z = 2 without offset. For accurate mass measurements, the lock mass option was enabled in MS mode.

Data were acquired using Xcalibur v2.5.5 and raw files were processed to generate peak list in Mascot generic format (*.mgf) using ProteoWizard release version 3.0.331. Database searches were performed using Mascot in-house version 2.4.0 to search the SwissProt database (Human, 20,279 proteins) assuming the digestion enzyme trypsin, at maximum one missed cleavage site, fragment ion mass tolerance of 0.05 Da, parent ion tolerance of 10 ppm, carbamidomethylation of cysteines as fixed modification, and phosphorylation of serines, threonines, and tyrosine, oxidation of methionines, and acetylation of the protein N-terminus as variable modifications. Scaffold (version Scaffold_4.4.8, Proteome Software Inc., Portland, OR) was used to validate MS/MS based peptide and protein identifications. Peptide identifications were accepted if they could be established at greater than 99.0% probability by the Scaffold Local FDR algorithm. Protein identifications were accepted if they could be established at greater than 99.0% probability and contained at least one identified peptide. The mass spectrometry proteomics data have been deposited to the ProteomeXchange Consortium via the PRIDE partner repository with the dataset identifier PXD014104 ^2^.

The criteria of phoshphopeptides, which were exclusively detected in monocytes or moDCs, was the detection of the peptide in all three biological replicates. The corresponding proteins of the phospho-peptides, which were exclusively detected in moDCs, was done using the Panther Classification System with the settings “GO:Slim Molecular Function”.

## Site-directed mutagenesis

Site-directed mutagenesis was performed on pDONR223 ARAF (Cat. No. 23725, Addgene) and pDONR223 CRAF (Cat. No. 23832, Addgene). The point mutations in ARAF (S257A, S257D) were generated using the *Pfu* Polymerase (Cat. No. 600250, Agilent Technologies), while CRAF double mutants (CRAF S296A/S301A and CRAF S296D/S301D) were generated using Q5® High-Fidelity DNA Polymerase (Cat. No. M0491S, New England Biolabs) by employing following primers:

hARAF S257A fw: 5’- gctggctgggctgggggccccccggggggttccatc-3’

rv: 5’-gatggaaccccccggggggcccccagcccagccagc-3’

hARAF S257D fw: 5'-ctgggctggggtccccccgggggg-3'

rv: 5'-ccccccggggggaccccagcccag-3'

hCRAF S296A fw: 5'-cagattgttgggggcactggacagggctgaagg-3'

rv: 5'-ccttcagccctgtccagtgcccccaacaatctg-3'

hCRAF S296D fw: 5'-ctcagattgttggggtcactggacagggctgaaggtg-3'

rv: 5'-caccttcagccctgtccagtgaccccaacaatctgag-3'

hCRAF S301A fw: 5'-gaccagcctgttggggccagattgttggggct-3'

rv: 5'-agccccaacaatctggccccaacaggctggtc-3

hCRAF S301D fw: 5'-gtgaccagcctgttgggtccagattgttggggctac-3'

rv: 5'-gtagccccaacaatctggacccaacaggctggtcac-3'

Subsequently, expression clones were generated in destination vector pcDNA3 Dest 40 (12274015, ThermoFisher) by using a Gateway® LR Clonase® II Enzyme mix (Cat. No. 11791-020, Thermo Fisher). Mutants were sequence verified.

## Cell culture

HeLa (DSMZ), Calu-1 (Sigma Aldrich), MDA-MB231 (DSMZ), HCT116 (a gift from Ulf Rapp) and HEK293T (a gift from Andreas Ernst) cell lines were cultured in Dulbecco’s Modified Eagle Medium (DMEM) (Cat. No. 11965092, Gibco) supplemented with 10% fetal calf serum (FCS) (Cat. No. 51437093, Life Technologies), while H226 (ATCC) cells were cultured in Roswell Park Memorial Institute (RPMI) medium (Cat. No. R8758, Gibco) containing 10% FCS. For experiments with the RAF inhibitor LY3009120, 2.5 x 10^5^ cells were seeded in 6-well plates, allowed to adhere overnight and subsequently treated with the inhibitor in starvation medium (RPMI without FCS) for 6 h. Cells were lysed either directly in RIPA buffer or after an additional stimulation with epidermal growth factor (EGF) for 5 min (100 ng/ml, Cat No. RP-10927, Thermo Fisher Scientific).

To investigate the effect of the MEK1/2 inhibitor trametinib and the RAF inhibitor LY3009120 on proliferation, the listed cell lines were seeded in 96-well plates at a density of 8 x 10^3^ cells per well, and on the next day, medium was changed to inhibitor-containing medium. Viability was analyzed after 72 h of treatment.

## Cell proliferation assay

Cell proliferation and viability was determined using the Cell Proliferation Kit I (MTT, Cat. No 11465007001 ROCHE, SIGMA-ALDRICH) following the manufacturer’s instructions.

## Transient transfection

For overexpression studies with the generated RAF mutants, 2.5 x 10^5^ HEK293T cells were seeded per well of a 6-well plate. Transfection with various plasmids was performed with polyethylenimine (PEI) (Cat. No. 23966, Polysciences Inc.) as transfection reagent. 1 µg DNA was transfected with 5.4 µl of 10 mM PEI. Analyses were performed 48 h after transfection.

## Transfection of siRNA

Immature, human moDCs were harvested and 0.6 x 10^6^ cells/well were seeded in a 12-well plate. To silence two RAF proteins at once siRNAs were combined with each other. The mixture of two siRNAs was transfected using SaintRed. Each siRNA was employed at a concentration of 60 nM. A scrambled control siRNA, which served as a negative control, was transfected at a final concentration of 120 nM. The medium was changed 24 h after transfection and moDCs were stimulated for 30 h with LPS (100 ng/ml). siRNAs were purchased from Qiagen:

siControl(sense): 5’-UUCUCCGAACGUGUCACGU-3’ (Cat. No. 1027310)

siARAF(sense): 5’-GACUCAAGGGACGAAA-3’ (Cat. No. SI00287686)

siBRAF(sense): 5’-GCUAGAUGCACUCCAACAATT -3’ (Cat. No. S102632945)

siCRAF(sense): 5’-GGAUGUUGAUGGUAGUACA-3’ (custom-made)

## Cycloheximide (CHX) chase assay

Changes in protein stability during the differentiation process from human monocytes to DCs were analyzed by a cycloheximide chase assay. The assay was performed on day 3 of culture and on day 5 of culture. Cycloheximide (Cat. No. C-7698, Sigma) was added (100 μg/ml) to the cells, which were lysed in RIPA buffer after 1 h, 2 h, 4 h and 6 h. Protein lysates were analyzed by SDS-PAGE and subsequent immunoblotting.

## Antibodies

Antibodies used in this study recognize human antigens. Phosphorylated CRAF at S338 (rabbit monoclonal, Cat. No. 9427S), total CRAF (rabbit polyclonal, Cat. No. 9422), total ARAF (rabbit polyclonal, Cat. No. 4432), phosphorylated MEK1/2 at S217/S221 (rabbit monoclonal, Cat. No. 9154S), total MEK1/2 (rabbit polyclonal, Cat. No. 9122), phosphorylated ERK1/2 at Tyr202/Tyr204 (rabbit polyclonal, Cat. No. 9101), total ERK1/2 (rabbit polyclonal, Cat. No. 9102) were purchased by Cell Signaling Technology. Total BRAF (mouse monoclonal, Cat. No. sc-5284) was obtained from Santa Cruz. The antibodies against the housekeeping genes GAPDH (mouse monoclonal, Cat. No. GTX627408) and Tubulin (mouse monoclonal, Cat. No. GTX628802) were bought from GeneTex. Anti-β Actin (mouse monoclonal, HRP-conjugated, Cat. No. ab49900) was purchased from Abcam and the monoclonal mouse V5 antibody was bought from Invitrogen (R960-25).

Immunoprecipitations of ARAF and CRAF were done with mouse monoclonal antibodies purchased from Santa Cruz and BD Transduction Labs (Cat. No. 610151). Mouse IgG1 antibody from Santa Cruz (sc3877) was used as IgG control in immunoprecipitation studies.

Antibodies for flow cytometry:

To characterize human moDCs, the following antibodies for flow cytometry were purchased from Biolegend: BV605 anti-human CD14 (Cat. No. 301833), BV605 mouse IgG2a (Cat. No. 400269), BV421 anti-human HLA-DR (Cat. No. 307635), BV421 mouse IgG2a (Cat. No. 400259), FITC anti-human CD80 (Cat. No. 305206), FITC mouse IgG1 (Cat. No. 400107), PerCP/Cy5.5 anti-human CD80 (Cat. No. 305231), PerCP/Cy5.5 mouse IgG1 (Cat. No. 400149), PE anti-human CCR7 (Cat. No. 353204), PE mouse IgG2a (Cat. No. 400213). PE anti-human CD86 (Cat. No. 555665), PE mouse IgG2b (Cat. No. 555743), APC anti-human CD83 (Cat. No. 551073), APC mouse IgG1 (Cat. No. 555751) were purchased from BD Bioscience.

The characterization of mouse bone marrow-derived DCs and of splenic DCs was performed with following antibodies (Biolegend): FITC anti-mouse CD11c (Cat. No. 117306), FITC Armenian hamster IgG (Cat. No. 400906), Pacific Blue anti-mouse I-A/I-E (Cat. No. 107620,), Pacific Blue™ Rat IgG2b (Cat. No. 400627), APC anti-mouse CD86 (Cat. No. 105012), APC Rat IgG2a (Cat. No. 400512), Brilliant Violet 605™ anti-mouse CD80 (Cat. No. 104729,), Brilliant Violet 605™ Armenian Hamster IgG (Cat. No. 400944), PE anti-mouse CCR7 (Cat. No. 120106), PE Rat IgG2a (Cat. No. 400508), BV510 anti mouse CD45 (Cat. No. 103137), BV510 rat IgG2b (Cat. No. 400645), PE anti-mouse CD11c (Cat. No. 117307), PE hamster IgG (Cat. No. 400907), FITC anti-mouse CD86 (Cat. No. 105109), FITC rat IgG2b (Cat. No. 400633), APC anti-mouse CD80 (Cat. No. 104714), APC hamster IgG (Cat. No. 400911).

## SDS-PAGE and Western Blotting

Cell lysates were prepared in sodium dodecyl sulfate (SDS) sample buffer (0.125 M Tris-HCl, pH 6.8, 4% SDS, 10% glycerol, 10 mM DTT and bromophenol blue) and loaded onto 7.5% polyacrylamide gels. The separated proteins were transferred onto nitrocellulose membranes (Cat. No. 10401296,Whatman Protran) using the wet/tank Blotting system from Bio-Rad. For immunoblot analysis, membranes were blocked with 3% BSA in PBS-T (PBS with 1% Triton X-100) for 1 h at room temperature. The incubation with primary antibodies was performed as suggested by the antibody providers. Horseradish peroxidase coupled secondary antibodies were then employed to visualize the antigen-antibody complexes by enhanced chemiluminescence (Cat. No. WBKLS0500, Millipore). Quantification of Western Blots was performed by densitometry (ImageJ software). Total protein levels were generally normalized to the levels of a housekeeping gene or to the Ponceau S staining of the entire membrane. Changes in total protein levels during moDC differentiation are shown in relation to levels detected in monocytes. The amount of phosphorylated protein was quantified by determining the ratio of phosphorylated protein to total protein levels.

**References**

1. Tran, T.T., Strozynski, M. & Thiede, B. Quantitative phosphoproteome analysis of cisplatin-induced apoptosis in Jurkat T cells. *Proteomics* **17** (2017).

2. Perez-Riverol, Y. *et al.* The PRIDE database and related tools and resources in 2019: improving support for quantification data. *Nucleic Acids Res* **47**, D442-D450 (2019).

**Supplementary Figure legends**

**Supplementary Fig. 1 Influence of CRAF Mutants on MAPK signaling. a,** The phosphorylation sites CRAF S296 and S301, identified in the phosphoproteome analysis and enriched in moDCs, were mutated to either alanine [A] or aspartic acid [D]. Double mutants were generated. CRAF mutants were transfected into HEK293T cells. Wild type CRAF and a kinase dead mutant (CRAF R401H) were included as a control. Phosphorylation of MEK1/2 (S217/221) was analyzed by immunoblot. **b,** ARAF WT and ARAF mutants (ARAF S257A and ARAF S257D) were overexpressed in HEK293T cells, immunoprecipitated via their V5 tag and kinase activity was studied as mentioned in the methods. Phosphorylation of the MEK K97A substrate at S217/221 was investigated by Western Blot. The kinase active mutant (ARAF Y301D/Y302D) and kinase dead mutant (ARAF R362H) were included as controls.

**Supplementary Fig. 2 Proteostasis of RAF proteins. a,** Differentiation of human monocytes was initiated by GM-CSF/IL-4 and after 3 and 5 days cells were treated with MG132 (10 μM) for 6 h. RAF protein levels were analyzed by Western Blot. **b,** The change in ARAF, **c,** BRAF and **d,** CRAF protein levels after MG132 treatment (10 µM, 6 h) was quantified (n = 3). **e,** Cells on day 3 and day 5 of differentiation were treated with 10 μM MG132 for 6 h. ARAF and CRAF were immunoprecipitated and analyzed by Western Blot.

**Supplementary Fig. 3, RAF, MEK and ERK activation during moDC differentiation. a,** The change in total MEK1/2 levels (refer to Figure 2 a) during moDC differentiation was quantified (n = 6). **b,** MEK1/2 (S217/221) (n = 6) and **c,** ERK1/2 (Y202/Y204) phosphorylation (n = 6) during the differentiation process was quantified by determining the ratio of phosphorylated protein to total protein (refer to Figure 2A). **d,** Representative Western Blot monitoring ARAF, BRAF and CRAF protein levels during the differentiation of murine BM cells to BMDCs induced by GM-CSF/IL-4.

**Supplementary Fig. 4 Double knockdown of RAF proteins in moDCs by siRNAs. a,** siRNAs were transfected to specifically knockdown RAF proteins in moDCs. A double knockdown of two RAF proteins was achieved by combining the corresponding siRNAs (siA+BRAF, siA+CRAF and siB+CRAF). LPS (100 ng/ml) stimulation was started 24 h after siRNA transfection in fresh medium. Knockdown of RAF proteins was confirmed by Western Blot analysis. **b,** Knockdown efficiency was determined after normalizing the RAF levels to the corresponding actin signal or Ponceau S staining. **c,** Surface marker expression of CD80 and CD83 (siA+BRAF and siB+CRAF: n = 3; siA+CRAF: n = 2) and **d,** cytokine secretion of IL-12p70 was analyzed 54 h after siRNA transfection including a 30 h stimulation with LPS (siA+BRAF: n = 2, siA+CRAF: n = 3; siB+CRAF: n = 4). (ND = not detected)

**Supplementary Fig. 5 MAPK signaling upon LY3009120 treatment.** **a,** The role of the MAPK cascade in DCs was investigated by employing the pan-RAF inhibitor LY3009120 and the MEK inhibitor trametinib. **b,** HeLa cells were treated with different LY3009120 concentrations ranging from 0.1 µM to 10 µM and MEK1/2 and ERK1/2 phosphorylation was investigated after 6 h treatment by Western Blot analysis **c,** Cancer cell lines (HeLa, Calu1, MDA-MB231) were treated with the RAF inhibitor LY3009120 (1 µM) for 6 h in starvation. Optionally, they were stimulated for 5 min with EGF (100 ng/ml). MEK (S217/221) and ERK (Y202/204) phosphorylation was investigated by Western Blot. **d,** Metabolic activity of cancer cells was measured by MTT assay after treating cells for 72h with either LY3009120 (1 µM) or trametinib (1 µM) (n = 3). **e,** Immature moDCs were treated for 6 h with LY3009120 (1 µM) or with the vehicle (DMSO). Kinase activity of CRAF and BRAF pulled down from the treated moDCs was determined by a kinase assay using MEK K97A as a substrate. Shown is a representative experiment.

**Supplementary Fig. 6 Effects of various RAF inhibitors and stimuli on the activation of moDCs.** **a,** MHCII and CD86 surface expression were analyzed by flow cytometry after 48 h treatment with trametinib (1 μM) or with LY3009120 (1 μM) in absence or presence of LPS (100 ng/ml). **b,** The relative mean fluorescence of the surface markers of multiple independent experiments was quantified. **c,** Immature moDCs were treated with increasing LY3009120 concentrations under LPS stimulating conditions and surface marker expression was analyzed 48 h after treatment. **d,** Complete list of all RAF inhibitors used in this study. **e,** Flow cytometer analysis of CD83 and CD80 after stimulating moDCs for 48 h with LPS (100 ng/ml), Poly(I:C) (50 µg/ml) or Pam3Cys (1 µg/ml)

**Supplementary Fig. 7 PGE_2_ in combination with LPS enhances CCR7 surface expression in moDCs.** CCR7 surface marker expression of immature moDCs (grey) and moDCs stimulated with LPS (100 ng/ml, green) or with a combination of LPS and PGE_2_ (PGE_2_: 1 μg/ml, red) was analyzed 48 h after treatment by flow cytometry.

**Supplementary Fig. 8 Flow cytometer analysis of murine DCs. a)** The expression of CD11c of BMDCs was checked by FACS analysis. **b)** Surface expression of CD80, CD86 and CCR7 of CD11c^+^ BMDCs was analyzed by flow cytometer after treating BMDCs with LY3009120 (1 μM) or trametinib (1 μM) in presence of LPS (100 ng/ml). **c)** Gating strategy to identify splenic DCs. Expression of CD86 and CD80 from CD11c^high^MHC^high^ cells was investigated. The histograms are representative for CD86 and CD80 expression of control mice (unstimulated) and LPS-treated mice.
