# Supplementary material for: RAF kinases are stabilized and required for dendritic cell differentiation and function
Source: Cell Death Differ. 2019 Sep 20;27(4):1300–15. doi: 10.1038/s41418-019-0416-4 (PMC7206131; doi:10.1038/s41418-019-0416-4)

Supplementary Figure 1

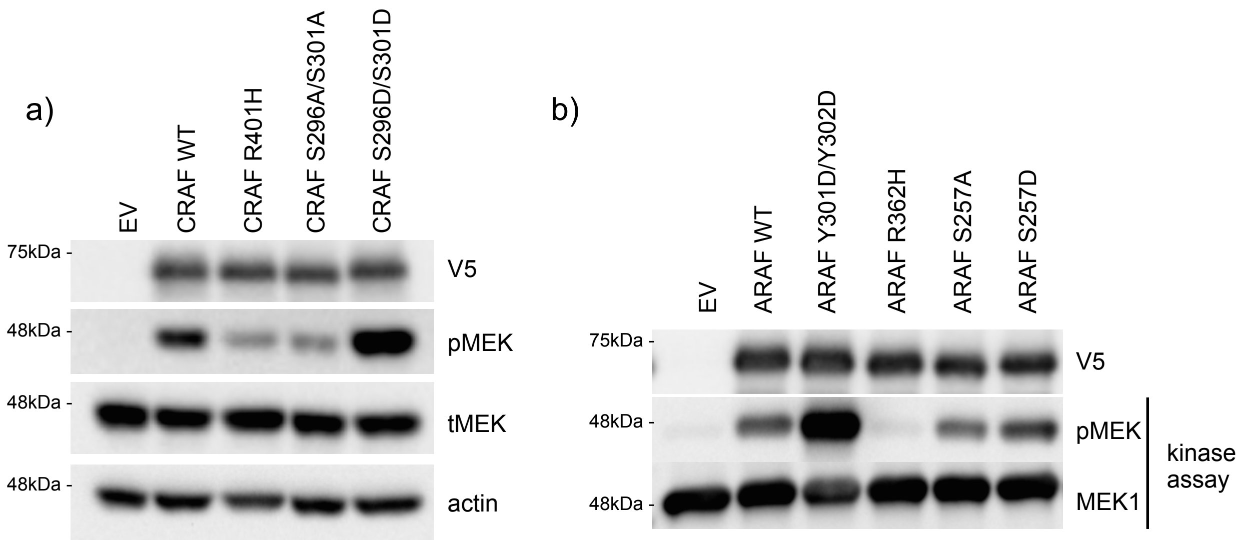

Supplementary Figure 2

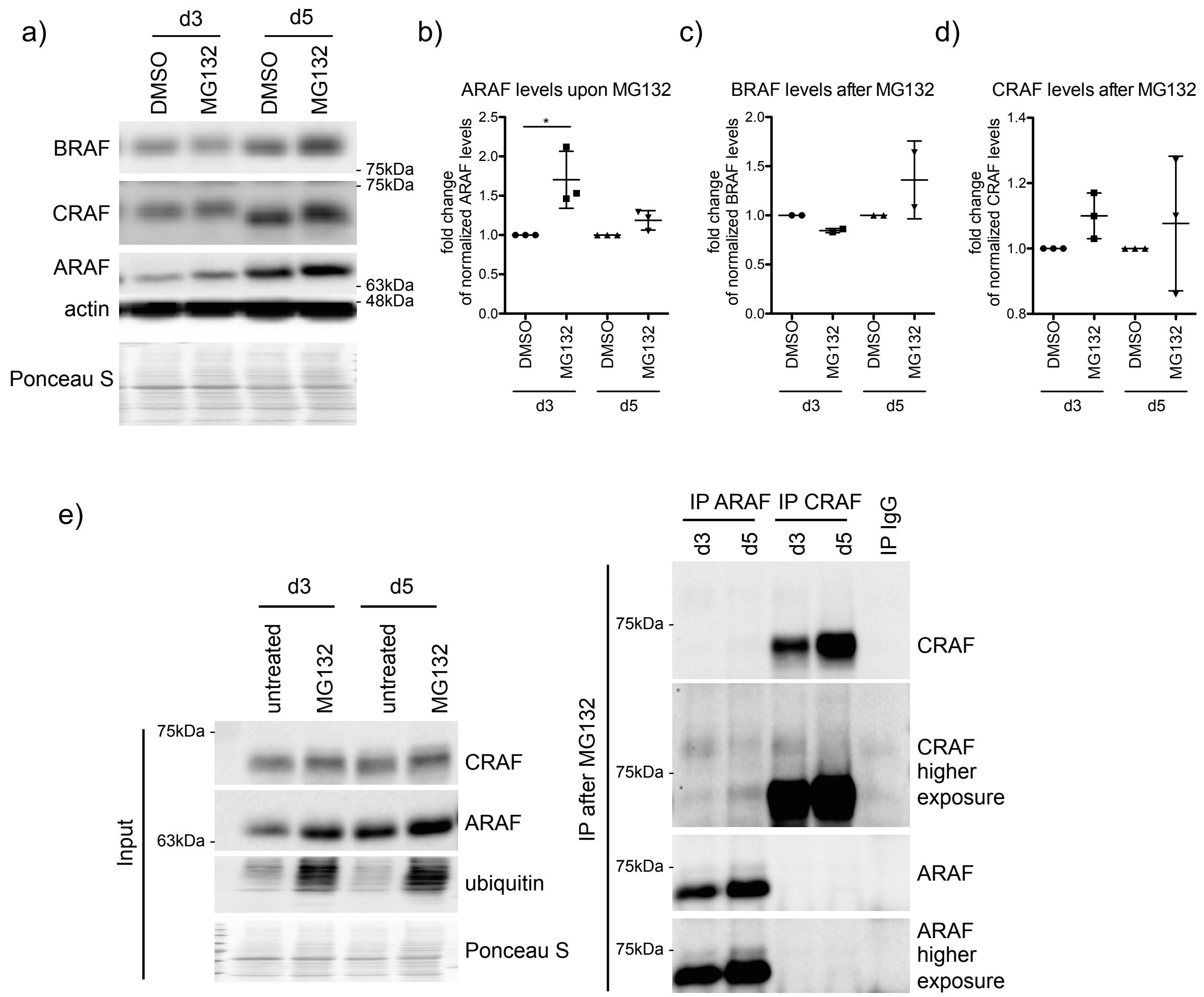

Supplementary Figure 3

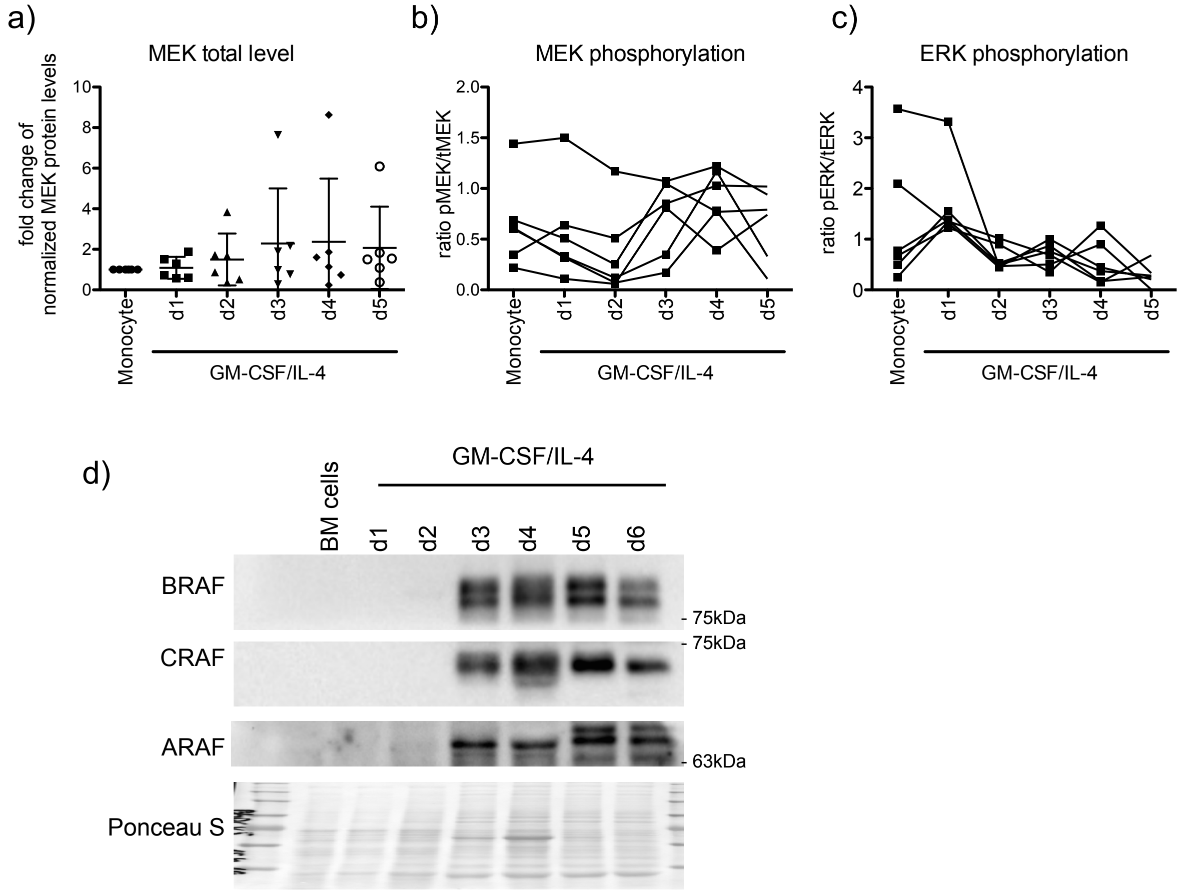

Supplementary Figure 4

a)

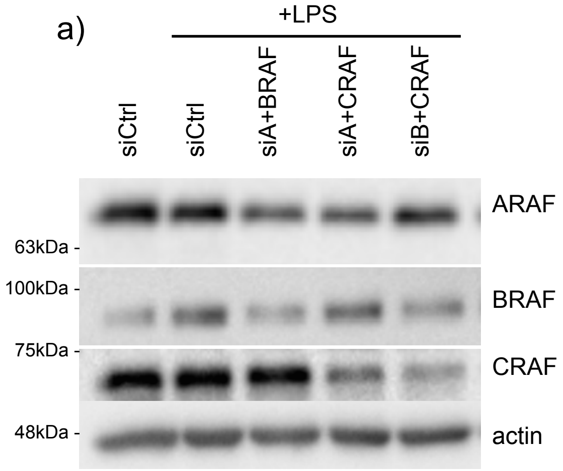

b)

knockdown efficiency in %

	siARAF	siBRAF	siCRAF
siA+BRAF	32-42%	34-45%	
siA+CRAF	40-82%		29-38%
siB+CRAF		21-57%	28-72%

c)

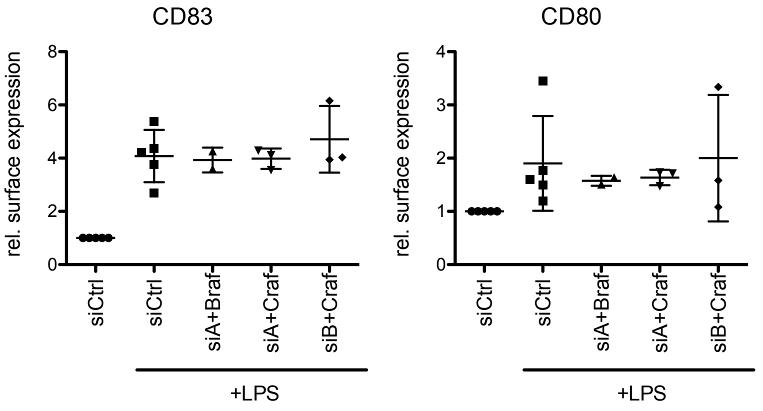

d)

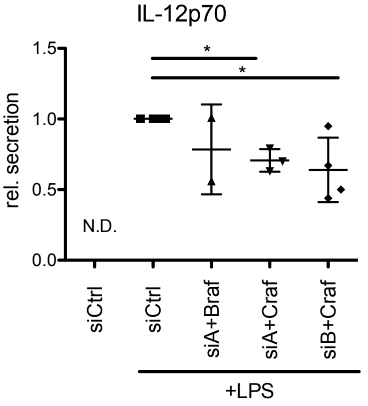

Supplementary Figure 5

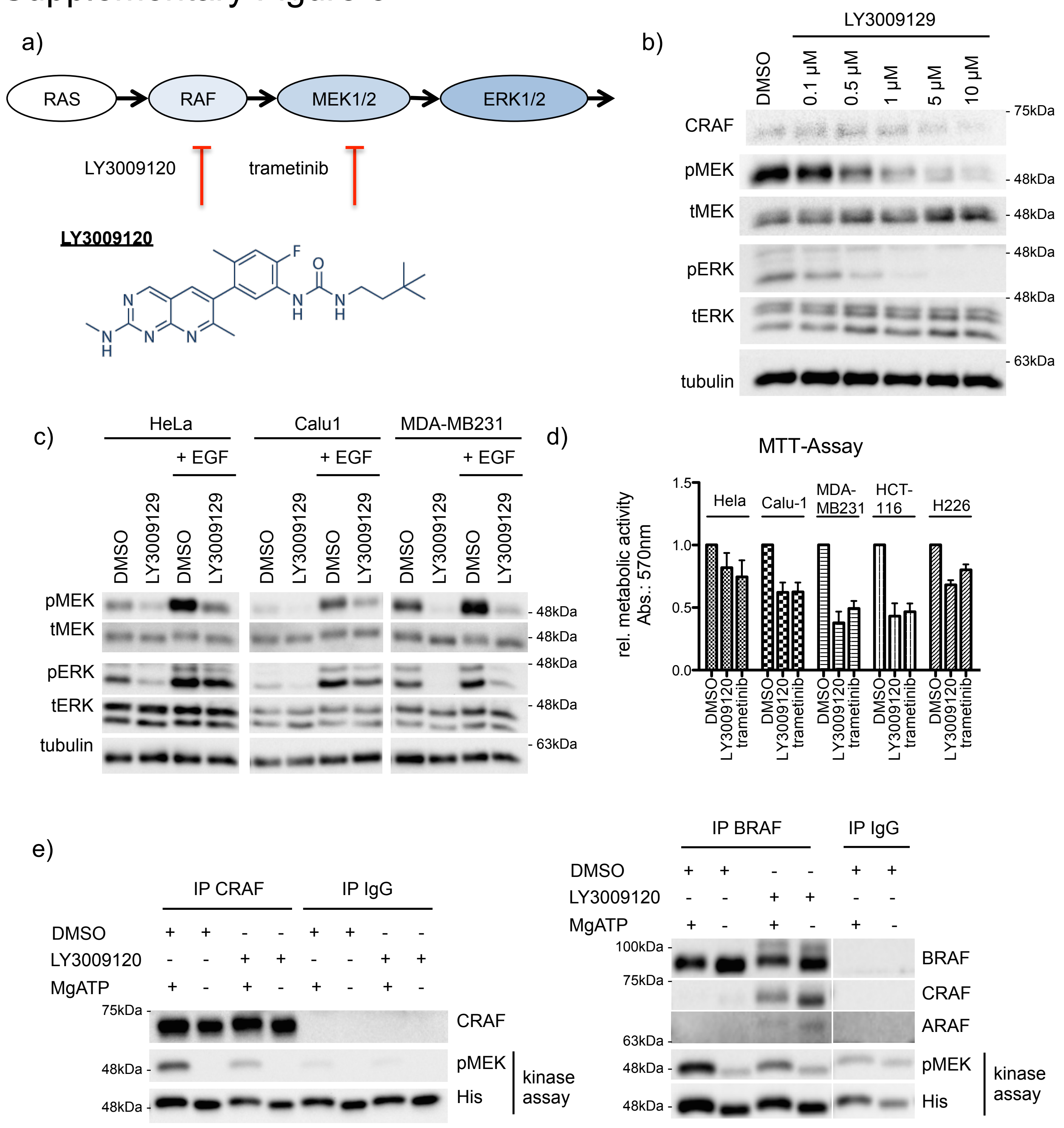

Supplementary Figure 6

a)

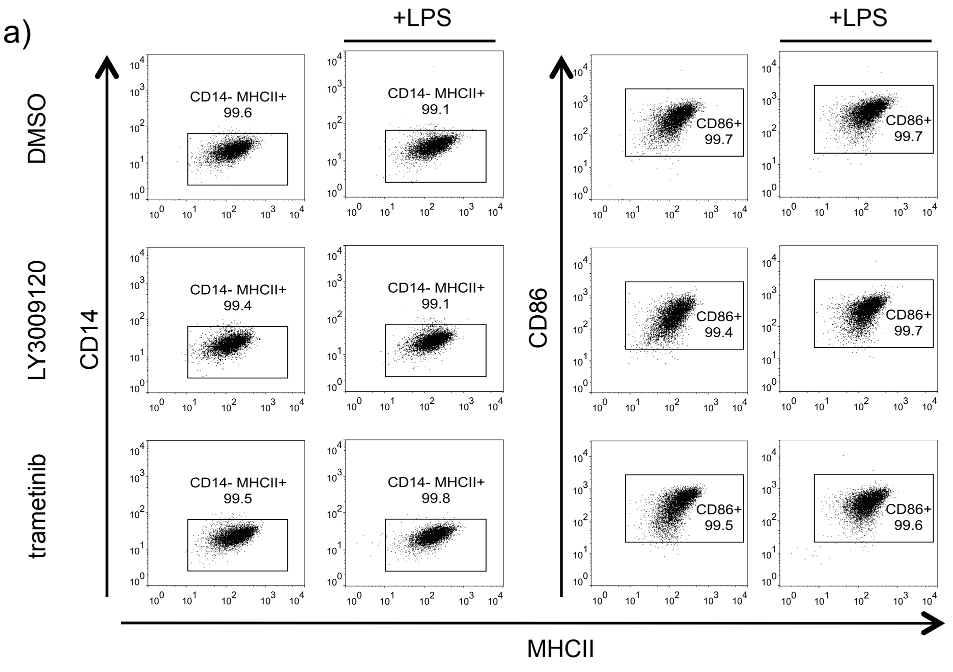

b)

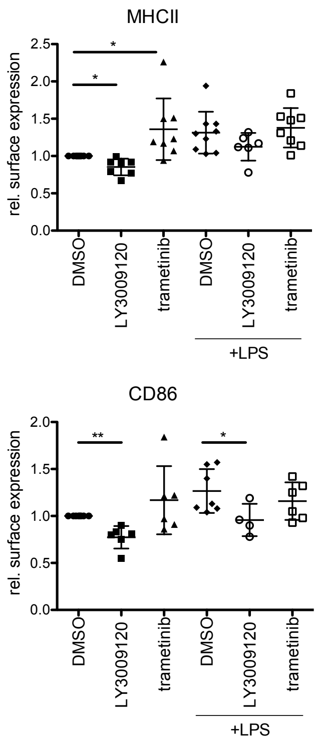

c)

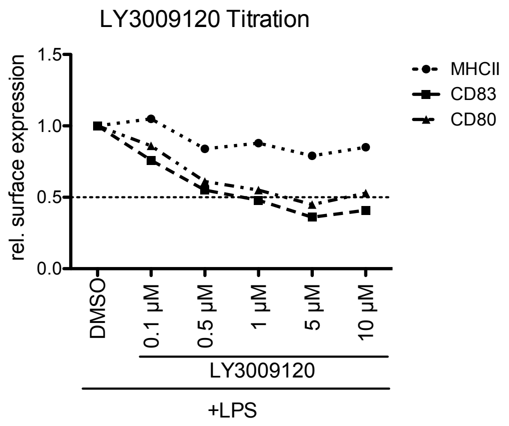

d)

Inhibitor	Mechanism
LY3009120	potent pan-RAF inhibitor for ARAF, BRAF, and CRAF
GDC-0879	selective BRAF inhibitor with activity against CRAF
Raf265	inhibitor of CRAF/BRAF/BRAF V600E , exhibits potent inhibition on VEGFR2
Kobe	H-RAS-CRAF interaction inhibitor
PLX4720	inhibitor of BRAF V600E, 10-fold selectivity for BRAF V600E than wild-type BRAF

e)

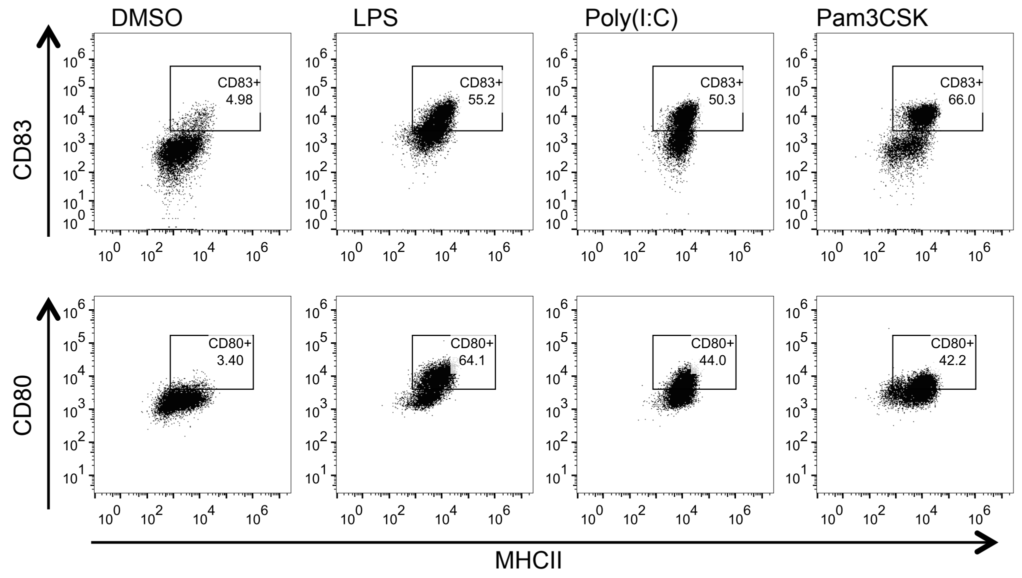

Supplementary Figure 7

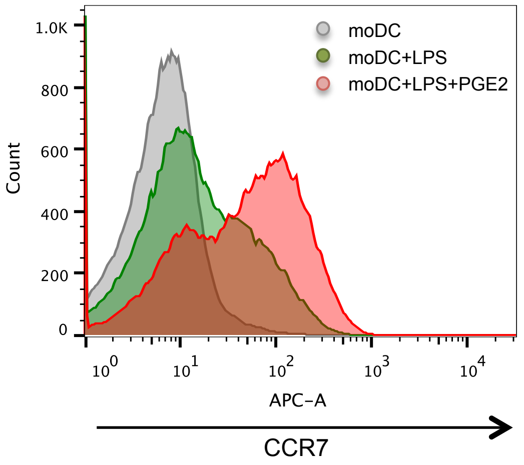

Supplementary Figure 8

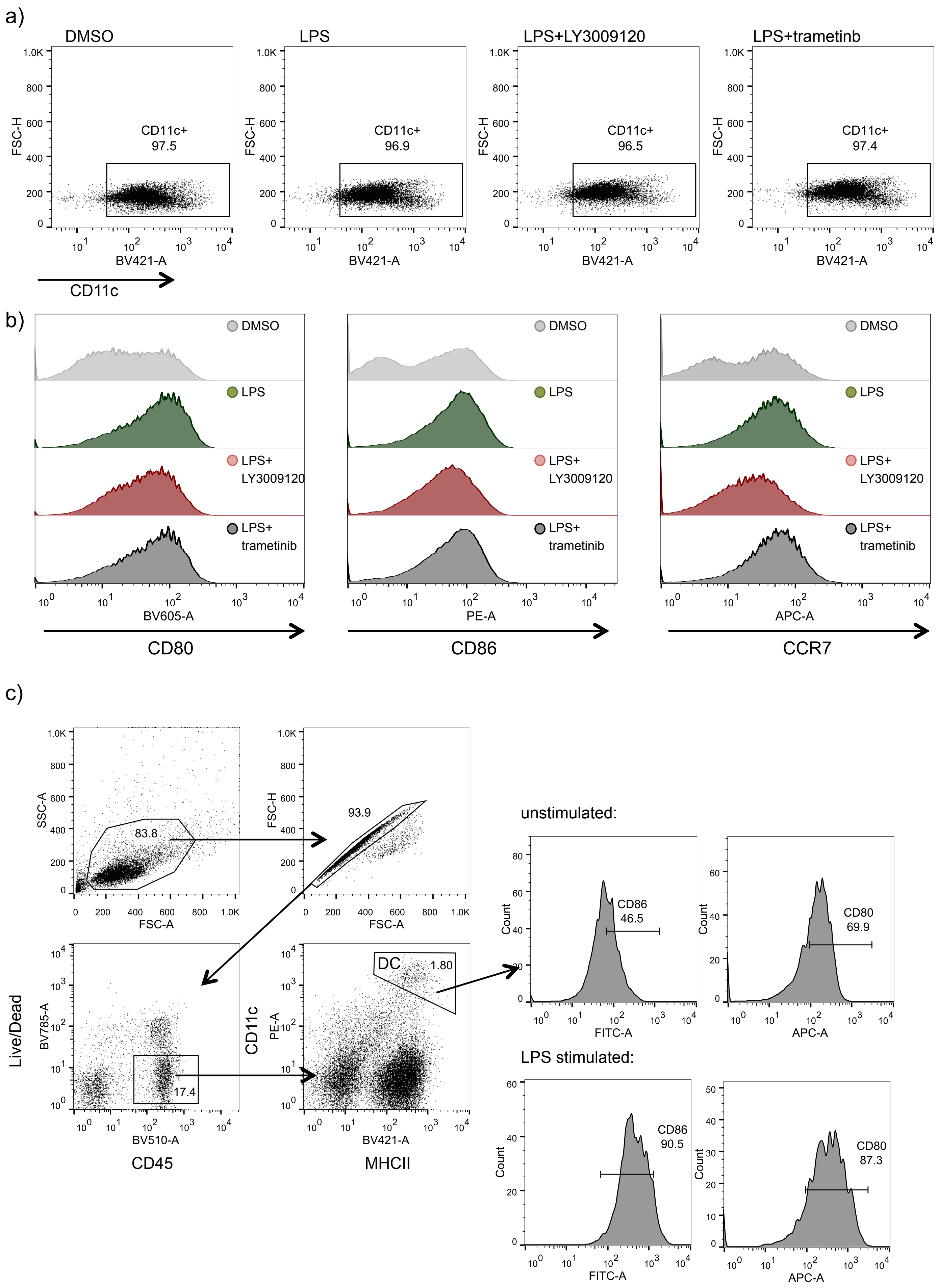

Supplement: Supplementary file 2 — Supplementary Figures [file 41418_2019_416_MOESM2_ESM.pdf]
